# DAISY: A Data Information System for accountability under the General Data Protection Regulation

**DOI:** 10.1093/gigascience/giz140

**Published:** 2019-12-04

**Authors:** Regina Becker, Pinar Alper, Valentin Grouès, Sandrine Munoz, Yohan Jarosz, Jacek Lebioda, Kavita Rege, Christophe Trefois, Venkata Satagopam, Reinhard Schneider

**Affiliations:** Luxembourg Centre for Systems Biomedicine, University of Luxembourg, Campus Belval 6, Avenue du Swing L-4367, Belvaux, Luxembourg

**Keywords:** GDPR, accountability, data mapping

## Abstract

**Background:**

The new European legislation on data protection, namely, the General Data Protection Regulation (GDPR), has introduced comprehensive requirements for the documentation about the processing of personal data as well as informing the data subjects of its use. GDPR’s accountability principle requires institutions, projects, and data hubs to document their data processings and demonstrate compliance with the GDPR. In response to this requirement, we see the emergence of commercial data-mapping tools, and institutions creating GDPR data register with such tools. One shortcoming of this approach is the genericity of tools, and their process-based model not capturing the project-based, collaborative nature of data processing in biomedical research.

**Findings:**

We have developed a software tool to allow research institutions to comply with the GDPR accountability requirement and map the sometimes very complex data flows in biomedical research. By analysing the transparency and record-keeping obligations of each GDPR principle, we observe that our tool effectively meets the accountability requirement.

**Conclusions:**

The GDPR is bringing data protection to center stage in research data management, necessitating dedicated tools, personnel, and processes. Our tool, DAISY, is tailored specifically for biomedical research and can help institutions in tackling the documentation challenge brought about by the GDPR. DAISY is made available as a free and open source tool on Github. DAISY is actively being used at the Luxembourg Centre for Systems Biomedicine and the ELIXIR-Luxembourg data hub.

## Background and Motivation

The General Data Protection Regulation (GDPR) [1] is a European Union (EU) regulation on data protection that is a directly applicable law in all EU member states since 25 May 2018. In addition, the GDPR has been incorporated into the European Economic Area (EEA) agreement and is therefore applicable to the member states of the European Free Trade Association as of 6 July 2018 [2]. In practice, however, the reach of the GDPR spans the whole world; where services or goods are being offered to citizens in the EU, the corresponding institution needs to comply with the GDPR, independent of where it is located. Furthermore, there is an obligation for data controllers in the EU to ensure that recipients outside the EU adhere to GDPR-equivalent data protection standards wherever possible. As a result of its global impact, the GDPR affects the international research community and has consequences for the way research data will be handled in the future [3].

One important new principle introduced by the GDPR in Article 5 is “accountability." Accountability requires the demonstration, and thus documentation, of the compliance with all data protection principles: lawfulness, fairness, transparency, purpose limitation, data minimization, accuracy, storage limitation, integrity, and confidentiality. While these principles were already the basis of the previous data protection directive [4], the necessity to record compliance is new. The documentation of the legitimacy of the processing including supporting information as well as of the processing are important requirements under the GDPR. Article 6 and Article 30 are key for these aspects. GDPR Article 6 outlines 6 possible “lawful bases” for processing personal data. Research institutions need to document under which basis their processings fall. For particular bases, such as “legitimate interest,” institutions need to perform assessments justifying this legal ground with “balancing tests” or “data protection impact assessments” [5, 6]. Article 30 of the GDPR outlines that institutions should record “data processings” and the records should at a minimum contain the following:

- Stakeholders, their roles (controller, processor), and contact information;
- Purposes of data processing;
- Categorical descriptions of data subjects and the data held on subjects;
- Categorical descriptions of recipients of data, particularly those in non-EU countries and international organizations;
- Logs of data transfers to identified recipients and a description of safeguards put in place;
- Where possible, time limits for keeping different categories of personal data;
- Where possible, descriptions of organizational and technical data protection measures.

GDPR requires that records of data processing should be auditable by supervisory authorities. Some countries such as Slovakia and Spain can even impose fines if these records are not in a suitable form for auditing [7, 8].

In biomedical research, data collected from human subjects and their biological specimens fall under the GDPR when such data are not anonymous. While GDPR’s accountability principle is often mentioned in the biomedical literature, we see that only a few publications discuss its implications, and then only briefly [9–11].

In response to the documentation requirement, we see the emergence of commercial “data mapping” tools, and academic institutions creating GDPR data registers with such tools [12, 13]. We observe that emerging approaches typically have 3 particular shortcomings for documenting the processing of personal data in biomedical research.

1. Documentation is typically done on a per-process basis. An institution’s activities, such as personnel administration or teaching, are recorded in terms of the processes involved, such as staff recruitment or student registration. When it comes to documenting research, however, the process-based approach falls short. Research is undertaken via projects, and each project represents a distinct report-worthy configuration in terms of what the GDPR roles of research collaborators are, what types of data are used, their de-identification status, and for how long the respective data can be kept.
2. Categorical descriptions of data subjects and data recipients do not provide sufficient transparency when handling data subjects’ requests for information. For example, Article 30 requires only the listing of categories of recipients with whom personal data have been shared, whereas Article 15 states that the data subject has the right to know the individual recipients or, if this is not possible, the categories of recipients. In addition, the source of the data needs to be provided on request as well as criteria for the retention of the data if no specific time limit is given.
3. A distinct characteristic of personal data processing in biomedical research is the separation of the collection of data from its continued processing. It is common to collect data from a cohort with a broad consent, as—due to the nature of research—possible uses cannot always be fully enumerated at the time of collection. In such cases, projects that collect, process, or further process data may be operating under different legal grounds (consent, public interest, legitimate interest), which need to be recorded to demonstrate compliance with Article 6 of the GDPR.

In this article we describe a software tool, named the Data Information System (DAISY), which can be used to meet the data documentation requirement that the GDPR imposes on biomedical research. The remainder of the article is organized as follows. We first introduce our method for data documentation and the target users of DAISY. Afterwards we discuss DAISY’s information model in detail and typical ways of inquiring this model. In Findings, we discuss how DAISY supports compliance with GDPR’s accountability principle. We conclude the article with final remarks and pointers to DAISY source code.

## Methods

DAISY has been developed to serve as a registry for research data processing that supports compliance with the GDPR and can serve for both auditing by the supervisory authorities and fulfilment of requests by data subjects.

**Figure 1:**
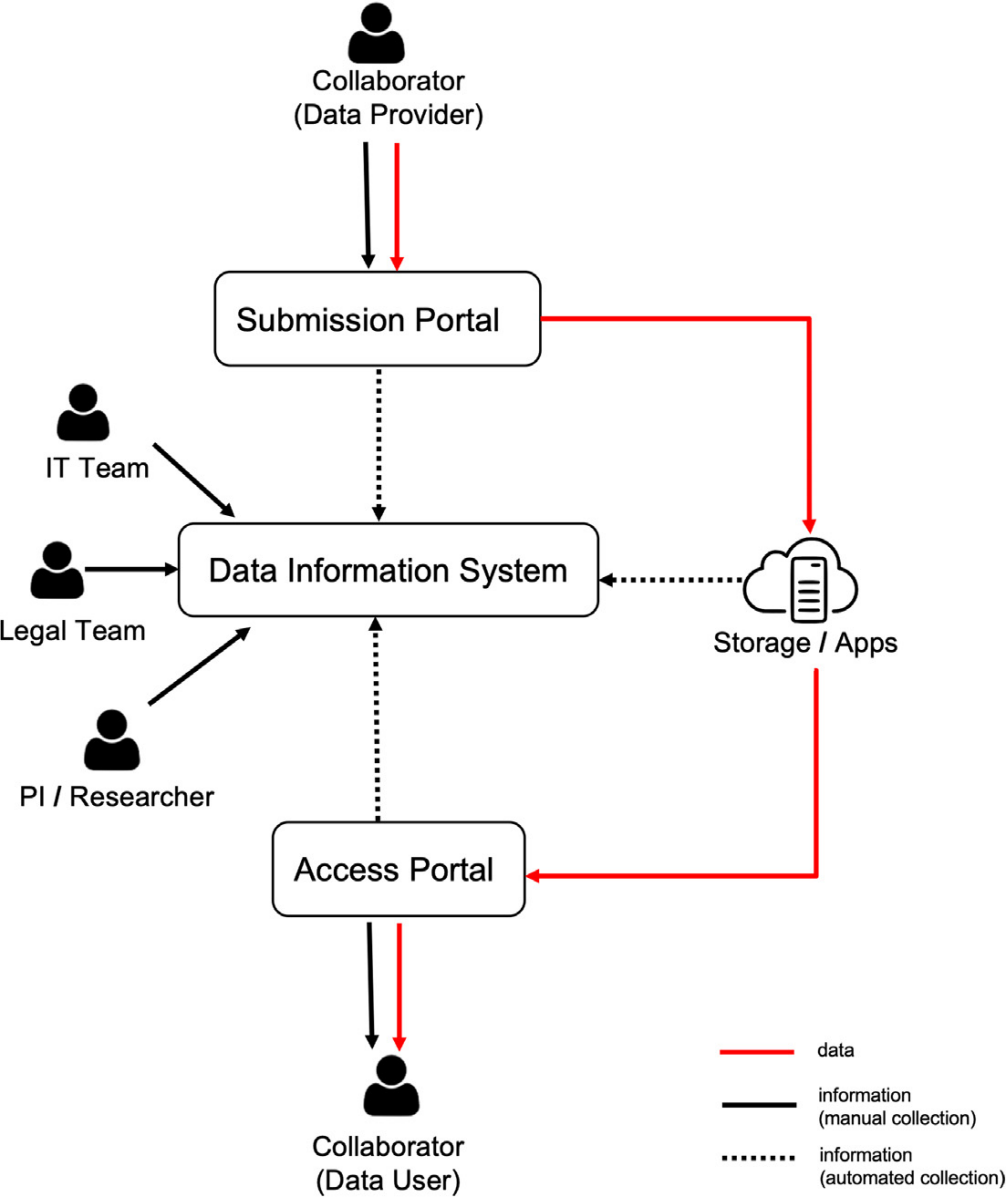
DAISY Target users and information flow.

During a GDPR audit, institutions need to provide information about what data they have and the lawful basis for data processing, as well as an appropriate record of the processing itself. To satisfy these requirements, one needs to pull together information from both human and machine sources (see Fig. 1).

Research principal investigators (PIs) have expert knowledge about the projects, the human biosamples, and data used as well as the collaborators who are the source or recipient of such data. The individual researchers know where and how data are held and processed. The legal department (Legal Team) establishes and maintains various kinds of contracts with collaborators and tracks the GDPR compatibility of those contracts. The information technology (IT) and systems administration personnel (IT Team) have an overview of what storage and transfer mechanisms are available for researchers, e.g., the submission portal where external collaborators can upload their data or the access portal where they can download or process data on site. The IT Team is also knowledgeable about the technical protection measures such as access control and encryption. Information from these stakeholders is typically distributed and in an unstructured form (e.g., email communication, documents, wiki/intranet).

In addition, the IT infrastructure and tools for data transfers, storage, and management should generate evidential information that can be used to demonstrate how data processing takes place in practice. Examples are time-stamped logs of user actions on data, granted or rejected accesses, or access permissions for a resource.

With DAISY, we initially focused on the manual collection of information from people through a web application developed with the Python programming language (RRID:SCR_008394) . The next phase of development will focus on plugins that can automatically harvest evidential data processing logs from IT infrastructure. In the following section, we outline the DAISY information model, and the possible inquiries that can be made over this model.

## DAISY Information Model

**Figure 2:**
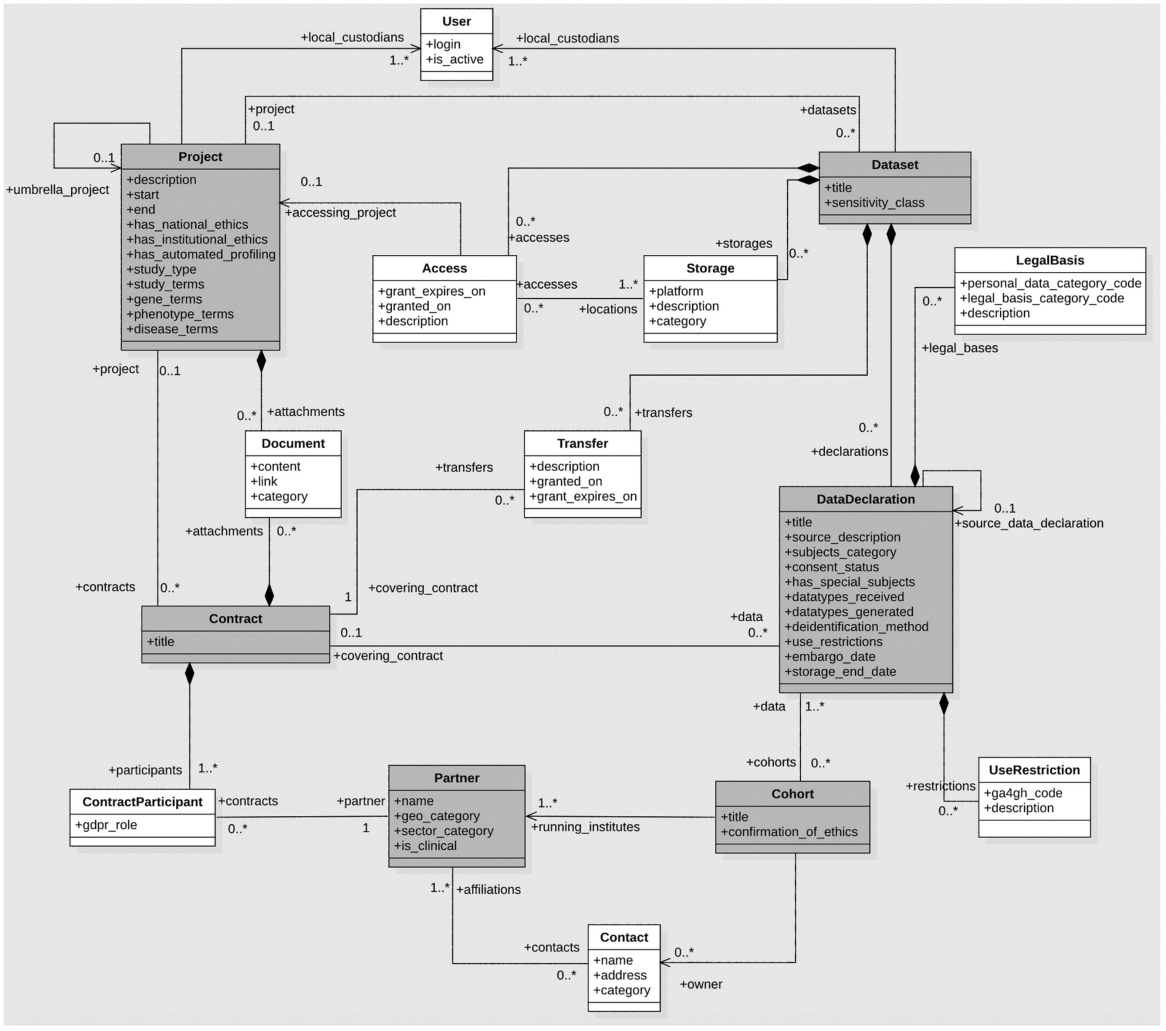
DAISY Information model.

At its core, DAISY is focused on recording research studies, the use of personal data of study participants, the sources of data, and the legal grounds for processing data. DAISY’s key entities are summarized in Table 1 for quick reference; the detailed model is given as a UML class diagram [14] in Fig. 2.

**Table 1:** Overview of key DAISY entities

Entity	Definition
Project	A time-limited research activity with associated documentation on the ethical, legal, and administrative procedures carrying out its implementation
Partner	A research collaborator who is the source and/or recipient of human data. Partners are also legal entities with whom contracts are signed. Clinical entities that run longitudinal cohorts, research institutes, or data hubs are examples of partners.
Contract	A legal agreement with one or more partners. Contracts are established by one or more mutually signed documents. Data-sharing agreements, consortium agreements, and material transfer agreements are examples of contracts.
Dataset	A physical/logical unit of data, which is typically treated as a resource with an associated location and access control policy
Data declaration	A sub-unit of data, which is traceable to a particular source, which could be the provider partner and source cohort or another data declaration
Cohort	A study that collects data and/or biosamples from a group of participants (e.g., longitudinal case-control or family studies). A cohort is linked to the creation of data and is considered its ultimate source.

"Project" represents a research activity occurring over a certain period of time. Projects can be organized in hierarchies; the long-term research programme of a laboratory can be recorded as an umbrella project and individual researchers’ work can be recorded as its sub-projects. DAISY allows projects to be tagged with controlled vocabulary terms to denote study features, as well as the study focus, such as diseases, genes, or phenotype attributes. The terms build, where possible, on standards; by default DAISY incorporates EDDA Study Designs Taxonomy [15], Human Phenotype Ontology [16], Human Disease Ontology [17], and Human Gene Nomenclature Symbols (HGNC) [18]. These can be replaced with ontologies of choice for a particular DAISY deployment.

Projects have one or more attached "documents." Documents are typically produced during the ethical, legal, and administrative procedures followed during project implementation. Documents can either be stored in DAISY, or they can also be linked to when they are archived in external document management systems. DAISY uses flags to track a project’s status on meeting national and institutional ethics requirements. In case of an absence of approvals, the user is expected to provide justifications. As per GDPR Article 22, it is necessary to track activities involving automated profiling of human subjects. An example of profiling in biomedical research is the calculation of disease ratings or scores from clinical attributes. DAISY flags such projects to enable reporting during audits. All stakeholders of a project including PIs, research, and administrative personnel can be recorded in DAISY. These could either be "contacts" in "partner" institutes or local personnel, which are recorded as users in DAISY. The primary responsible person for the GDPR documentation of human data used in research is the PI, and these users are marked as local custodians of projects and data.

"Contract" is another key category in DAISY used for data provenance tracking of incoming or outgoing data. A contract is a legal agreement with one or more signing partners, and it is typically associated with one or more legal "documents."

A project’s data are recorded as a "dataset" containing one or more "data declarations." A dataset is a physical or logical unit of data, which is typically treated as a resource (e.g., a folder of files, a database, or a laboratory book) with an associated storage end point and access control policy. Meanwhile, a data declaration represents the smallest unit of data that is traceable to a distinct data source. Data can be declared in the following 3 ways, depending on their source:

- Data that are being obtained from a partner (collaborator or repository) for the purposes of a project. These declarations will point to the source contract with the partner.
- Data that have been obtained in an earlier project and are being re-used in a follow-on project. These declarations will point to an existing data declaration as their source.
- Data that have not been obtained from a collaboration partner or an earlier project, such as data downloaded from community repositories. These declarations will have a source description in free text.

In addition to the above information on the data’s source, DAISY facilitates the maintenance of a list of "cohorts" and links data declarations with source cohorts. A cohort represents a study that collects data and/or biosamples from a group of participants (e.g., longitudinal case-control or family studies). With cohort annotations on data declarations, one can refer to all data that were generated around a group of data subjects. This is especially useful for the effective handling of a subject’s requests. For instance, when a subject withdraws consent or exercises his/her right of access, the affected projects and data can be easily identified using cohort annotations.

DAISY tracks "legal bases" of data processing and "use restrictions" per data declaration. Each data declaration can be associated with one or more legal basis definitions, which in turn is a combination of lawful basis category, out of 6 as defined by the GDPR, and personal data category, i.e., health, genetic, standard personal data, etc., as outlined by the GDPR. Even where consent is not chosen as the legal basis, in most cases ethical consent for the research itself is sought and determines use conditions that should be seen as much as possible as binding for the continued processing.

Within 1 cohort, subject consents and the resulting data processing permissions given by each subject may differ. We model this using a consent status flag on data declarations. This field denotes whether subject consents are heterogeneous or homogeneous. Data declarations with homogeneous consent would have a single use restriction configuration; meanwhile, those with heterogeneous consent have multiple use restriction configurations. The use restriction entity in DAISY models a restriction using a combination of the machine-readable “consent code” established by the Global Alliance for Genomics and Health (GA4GH) [19] and free-text descriptions. Examples of consent codes are project or disease or research area–specific restrictions on data use. DAISY actively monitors time-dependent restrictions such as embargo periods or storage durations and sends date expiry notifications to responsible users. GDPR puts special emphasis on data of “special subjects” such as minors or those unable to give consent. DAISY also allows the flagging of special subjects and documenting subject categories (e.g., cases, controls). For each data declaration, DAISY requires the recording of data types received, e.g., biosamples received, or metabolomics and whole-exome data generated.

DAISY records data processings, such as data access, storage, and third-party transfer, at the level of "datasets." For "storage", DAISY allows the definition of a set of platforms on which data could possibly be stored. These can be applications, relational databases, or file systems. This is relevant for the technical safeguards that go along with the respective platform. Storage is then defined in terms of a platform and a textual description e.g., actual link to the data. The policy on how local stakeholders, e.g., researchers or IT personnel, can access a dataset is captured with the "access" entity. Access definitions refer to storage locations, together with a free-text description of the access control policy and its validity period. "Transfer" of a dataset to external parties (EU/non-EU) is recorded with its time stamp, the recipient partner, the contract that establishes the legal basis of transfer, and a free-text description of safeguards taken for the transfer. Such safeguards are an obligation for third-country data sharing under the GDPR to ensure that the rights of the data subject are retained also under other data protection legislations.

Human data in research can be of varying sensitivity, and correspondingly varying protective measures are needed. For instance, whole-genome data, when accompanied with disease phenotypes, are considered highly sensitive and should be kept encrypted at rest [20]. During a GDPR audit, the handling of such data can be a particular focus. To support such inquiries, DAISY allows for defining data sensitivity classes that can flag data of higher sensitivity requiring additional safeguards and the tagging of datasets with those classes.

## Inquiring DAISY

**Figure 3:**
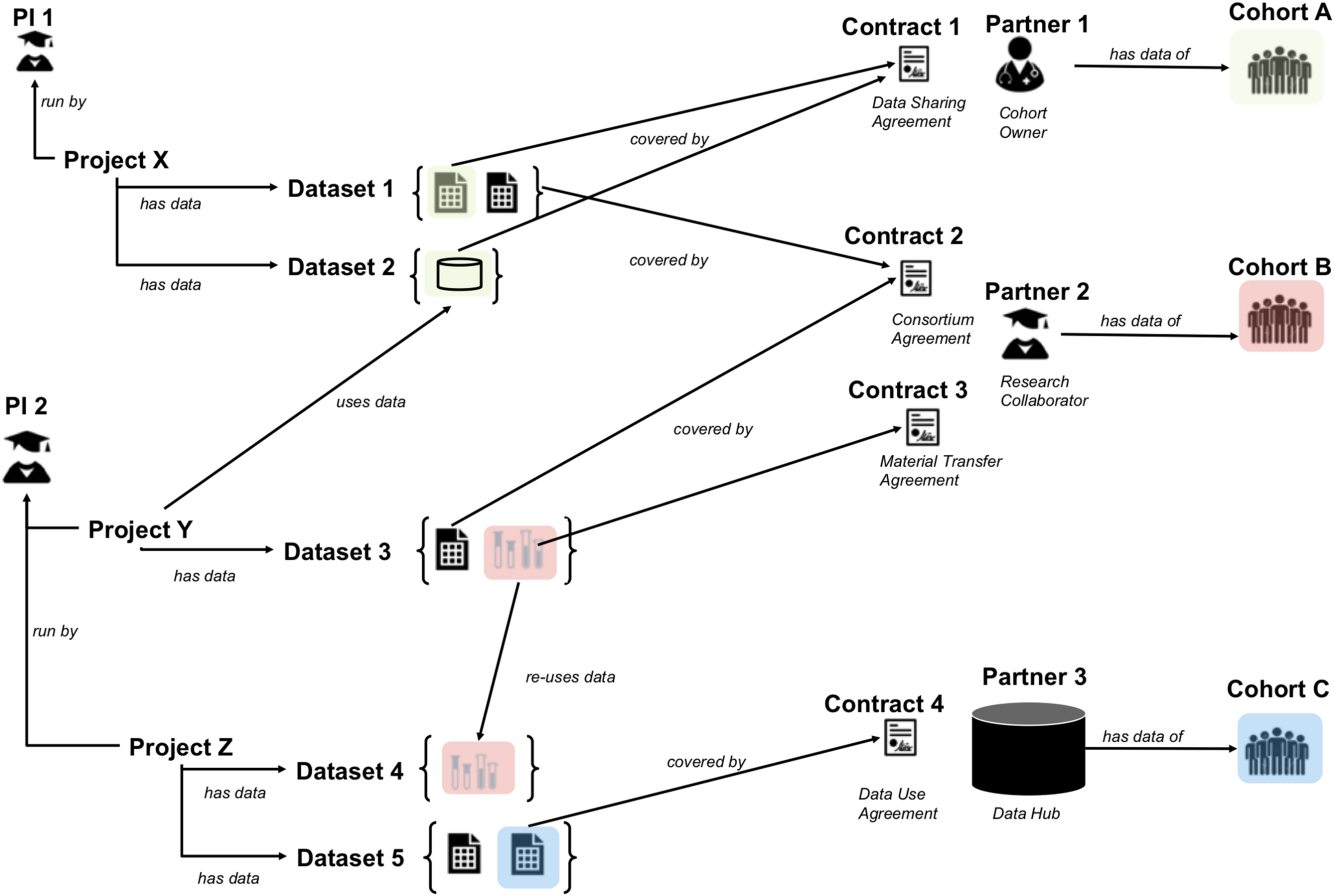
Example DAISY records and the relationships between them. Projects and datasets create a matrix of associations. A project may involve several datasets with (sub)data declarations coming from different source cohorts and covered by different contracts with various partners, which may have been signed at different times. Additionally, data/biosamples obtained for 1 project may be re-used in another project (biosamples in Dataset 3 of Project Y are re-used in Project Z Dataset 4). Furthermore, a project may access data of other projects (Project Y and Dataset 2). Typically, cohort data are collected for an open number of projects. Therefore, biosamples and data of 1 cohort may end up in several datasets within an institution; we denote this with colours: data from a specific cohort are shaded with the colour of that cohort. Also, note that researchers may generate different data types using biosamples/data obtained from a cohort, e.g., generate genomics data from samples. DAISY ensures that cohort annotations are propagated to newly generated (derivative) data.

A sample depiction of relations among DAISY entities is given in Figure 3. DAISY information space is commonly explored from 3 perspectives, based on datasets, projects, and contracts.

1. Datasets can be located on host storage platforms based on cohorts covered or the types of data contained. From here the user can navigate to the projects that (re)use these data as well as their sources and the contracts and documents ensuring the data’s legal and ethical processing.
2. Projects are indexed with their responsible PIs and are classified with respect to subject areas and study methods. From a project, one can navigate to datasets that are used in the scope of this project, including those made accessible from other projects. Projects also link to contracts and associated partners that have sent or received data.
3. Partners are indexed according to their geographic location, sector, and activity, e.g., as a healthcare institution. It is possible to get an overview of contracts signed with designated partners. From 1 contract, e.g., a consortium agreement, one can navigate to the encompassing project and then to other contracts such as material transfer or data-sharing agreements.

**Figure 4:**
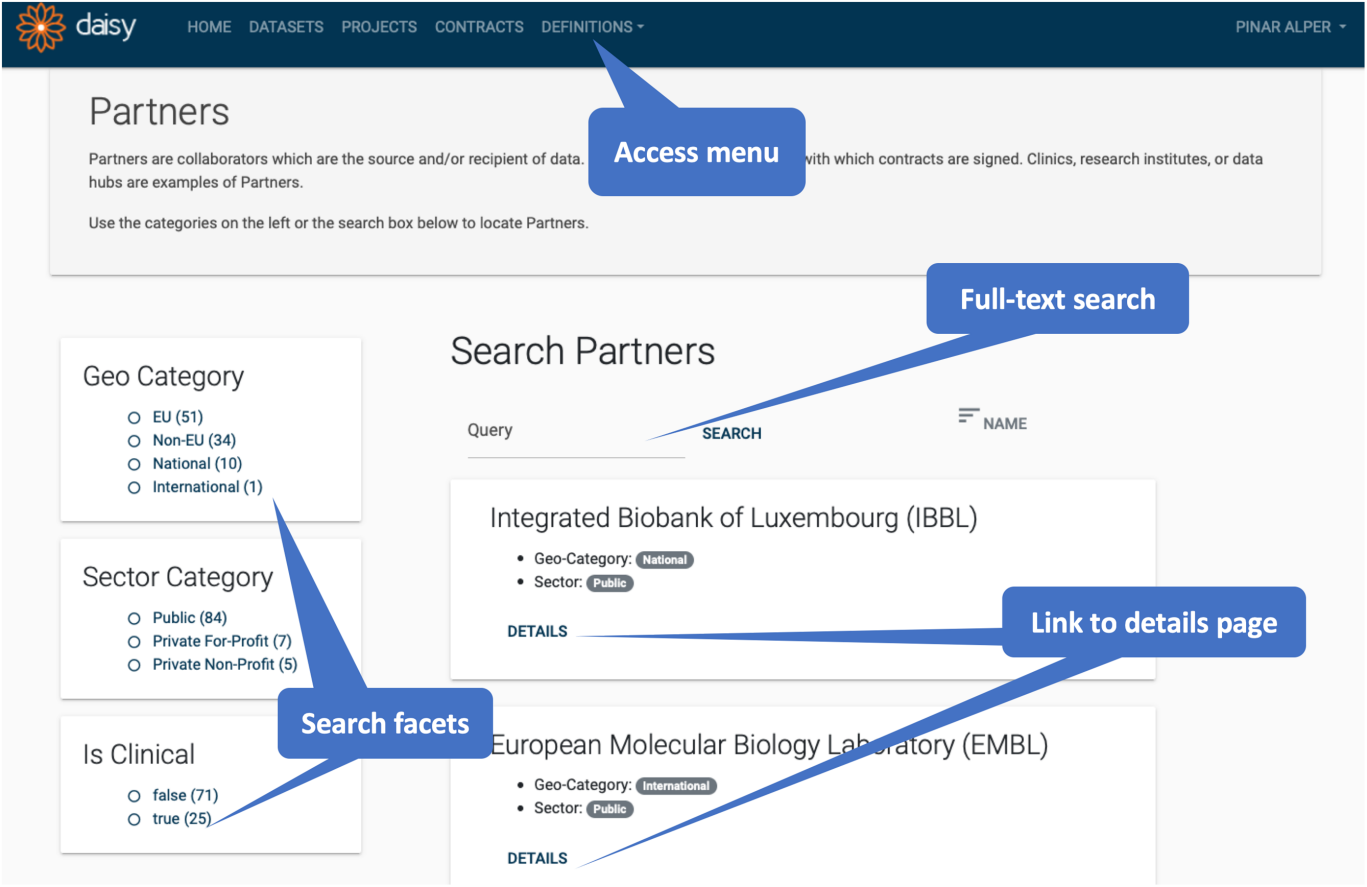
Screenshot of Partner search page in DAISY.

All inquiries start by locating records of interest and navigating to other related records. DAISY provides faceted search pages for all major entities and allows the traversal of links between entities. A screenshot of the partner search interface is given in Fig. 4.

## Findings

At its core, DAISY needs to provide all the information required to fulfil the transparency and record-keeping obligations of Articles 15 (right of access) and 30 (records of processing activities). However, for accountability, by following Article 5, further documentation is needed as described below. This combined information fulfils the requirements for accountability in the following way.

### Lawfulness

The legal basis for the processing is a mandatory field in DAISY. Additional entries enable either a reference to be made to external files or the upload of files that can further corroborate the chosen legal basis. In the case of legitimate interest, the balancing exercise will be needed. For consent as legal basis, the consent template is relevant. When the public interest basis is used, the reference to the respective law required following GDPR Article 6(3) should be provided. The legal basis is not a mandatory element of the record keeping in Article 30; however, it is crucial to document it to properly comply with the GDPR.

### Fairness

Documenting the fairness is a challenging undertaking. As an example, the UK Data Protection Act [21] states in Article 19(2) that the research must not cause substantial damage or substantial distress to a data subject. Within DAISY, the fairness principle for a clinical study is covered by the reference to, or the upload of, the participant information sheet and the corresponding consent form. These documents demonstrate the agreement of the study participants with the intended research. In addition, the ethics approval documents give an independent assessment that the purposes of the research do not override the interests of the study participants in an unfair way. The approach to prove fairness through an ethics approval is in some countries even part of the legislation, e.g., in the UK [21] or in Sweden [22].

Where data are imported from repositories and collaborators, the confirmation of the existence, and the coverage of, the ethics approval is obtained as part of the data-sharing agreement. This document also determines the purposes for which the data can be used in compliance with the fairness principle. In this case, no consent forms are needed and the ethics approval documents uploaded in DAISY will cover only the research project using the data.

### Transparency

The fields in DAISY cover all information requirements about the processing towards the data subject according to Articles 13, 14, and 15. The purpose of the field "project description" is to provide a lay summary that is understandable for the data subject. In addition, the record keeping following Article 30 is addressed within DAISY when the information is specific for the particular project. The more generic information on the technical and organizational security measures has to be documented outside DAISY (see below under confidentiality).

### Purpose limitation

The overall purpose of processing data for research is inherent for all research projects using the data. However, often the data have been collected for more specific reasons. Here, the scope of the consent of the study participants is the guiding principle, even if the chosen legal basis is not consent but, e.g., public interest or legitimate interest. Data collection in the medical research context requires a consent, wherever possible, from the ethics point of view, independent of data protection law [23–26]. Therefore, each dataset has a purpose limitation field that follows the machine-readable GA4GH codification [19]. This enables automated consent management to be performed at the study level. In addition, the "consent status" field specifies whether the obtained consent has been homogeneous or heterogeneous. Because the GDPR requires the consent to be given for each purpose individually [27], studies often offer a selective option to consent to the specific study in which context the data are collected, as well as additional future research projects, thus giving a broad consent for the data use in research. DAISY has a field to store the column(s) in the tabular clinical information that specifies the different consent options. With this, information about the position in the clinical data of the participants’ consent can be found and used for subject-level consent management. This enables an implementation of the purpose limitation at the level of individual subjects.

### Data minimization

Data minimization needs to be implemented on 2 levels, the administrative data within DAISY and the research data administered by DAISY. On the administrative side, DAISY foresees a role definition of different stakeholders with corresponding access rights (profile based). To allow data minimization of research data, DAISY lists the different types of data available in the datasets. This list gives input for the data analysis plan that should be uploaded as part of the project description and thus can provide the basis for a selective access provision.

### Accuracy

Information related to the data accuracy for the research data can be provided by linking to a metadata file or a management system that contains additional information on the data collected and standard operating procedures used. The metadata link can be found in the source project that describes the study or origin that led to the creation of the dataset. The accuracy of the administrative data of DAISY is to be achieved by the different access rights of the stakeholders editing DAISY. Some information, e.g., relevant legal data, can only be edited or changed by a legal administrator to avoid erroneous entries. In addition, record updates in DAISY are logged; the time stamp of updates and the user performing the operation are recorded.

### Storage limitation

The retention time for a dataset is an entry in DAISY. An active alerting system informs the responsible PI as well as the legal team and the data steward before the foreseen storage duration comes to an end.

### Integrity

The data integrity can be followed up through the creation of the checksum [28, 29] value that stays with the dataset during its lifetime from the ingestion into the IT infrastructure until its deletion. While the checksum is usually kept together with the respective dataset, it can also be saved within DAISY to keep track of it. The checksum value can be repeatedly tested to avoid silent corruption of the data and can be transmitted with each data transfer to ensure that no data are corrupted during transit.

In addition to data, another important aspect is the integrity and trustworthiness of metadata, i.e., the records in DAISY. Currently DAISY logs all database queries and manipulations, and enforces access control on those operations; but for its records to be used in compliance assessment, more assurances, similar to those provided by Code of Federal Regulation (CFR) 21 Part 11 compliant software, would be necessary [30]. The GDPR is yet to be translated into concrete industry guidance similar to CFR 21 Part 11; nevertheless, in DAISY we plan to implement queryable audit trails of records and record fixity and signing features. DAISY’s source code and user manual are tracked in Github; any changes to DAISY source go through a process of peer review; approval source changes are fully recorded; and source build and test procedures are automated. DAISY should be deployed, tested, and managed in protected environments respecting, where applicable, systems support standards and industry best practices, as those in the financial or the pharmaceutical sectors.

### Confidentiality

Both data controller and processor are obliged to implement appropriate technical and organizational measures to ensure the privacy of the data subjects (GDPR Articles 24–26 and 32). The organizational and technical security measures need to be documented following Article 30 of the GDPR. Rather than documenting all security measures directly in DAISY, a link to such external documentation is foreseen. Likewise, part of each project characterization in DAISY is a link to a data protection impact assessment that is performed externally. Currently DAISY stores URL links to external documents without checking for erasure or update of those documents. The linking feature is intentional because DAISY is typically co-deployed with document management systems dedicated to the storage of legal documents. We consider such external document management systems to be primarily responsible for preservation of documents and their revision tracking.

### National differences

Ultimately, full compliance with data protection law within the EEA may be subject to national or regional differences depending on the interpretation by the data protection authorities and the GDPR implementations in the different countries through local laws. In particular in the areas of research and health or genetic data, the GDPR leaves considerable scope for national provisions (see, e.g., [31]). Here, not only data protection law may be relevant but also subsequent sector laws regulating areas such as healthcare, biobanking, or research where additional requirements can be defined. Therefore, it is necessary for all data controllers to be aware of the relevant legislation in their countries. Data protection officers in the institutions can help in this quest. Where the requirements are not clear, the relevant data protection authority can be requested to provide clarification.

## Conclusion

DAISY can assist research institutions with a guidance on their documentation obligations and can serve as a knowledge base about the data existing in the institution. DAISY not only supports documentation towards accountability but also helps to comply with the responsibility principle as it warns, for instance, about the expiry of data retention times. It also gives indications about what further research is compatible with the existing consent by matching information on potential future projects with the consent codes stored for the data.

While the extensive documentation required may seem excessive, the GDPR actually puts in place what had already been postulated many years ago for health databases from an ethical point of view, such as the World Medical Association declaration on ethical considerations on health databases and biobanks [32]. With audits and the corresponding fines for non-compliance, the GDPR now enforces such ethical principles.

Because the documentation obligations described above are a new requirement under the GDPR, not many research institutions are ready for this administrative challenge. A particular hurdle is that for all personal data acquired prior to 25 May 2018, the same documentation is needed if the data are still being processed. This includes the mere storage of personal data. Substantial catching up will be needed.

The GDPR is leading to new positions in the research environment: as per Article 37, all public institutions as well as institutions processing sensitive data on a large scale are required to have a data protection officer. The administrative and documentation efforts around the data processing may also lead to increased employment of data stewards who ensure the proper ingestion and registration of data in systems such as DAISY. Such developments driven by data protection law may, however, also improve the quality of the data as the support given by data stewards can also lead to better documentation of scientific metadata and better structuring of databases. Data protection will ultimately become part of a research institution strategy in their efforts to make data FAIR: findable, accessible, interoperable, and reusable [33]. DAISY was developed as a tool to support this work.

## Availability of Source Code and Requirements

DAISY has been developed as a Python web application. Bootstrap and jQuery are used for the front end (HTML, CSS, Javascript). PostgreSQL is used as the database backend, and SolR is used as the full-text search engine. The DAISY source code is available online [34] with a GNU Affero General Public License (AGPL). DAISY installation has primarily been tested on Linux CentOS 7 servers; DAISY also has a simpler Docker-based deployment, which can be used for demonstration deployments and software evaluation. Instructions for both deployments can be found in our source repository [34].

DAISY has interfaces to allow the upload of JSON or Excel files to import relevant data from other information systems or following the independent collection of information. Also, a planned feature is to have exports of data provenance information in DAISY. Such exports can support requests for information during data protection audits or when responding to study participants’ requests.

DAISY has been registered in bio.tools [35] with the identifier biotools:Data_Information_System_DAISY,and in SciCrunch [36] with the identifier RRID:SCR_017472.

Project name: Data Information System

Project home page: https://github.com/elixir-luxembourg/daisy

Operating system(s): Platform independent

Programming language: Python 3.6 or higher

Other requirements: Solr 7.x, PostgreSQL 10.x

License: AGPL-3.0

RRID: SCR_017472

## Availability of Supporting Data and Materials

Snapshots of the code are available in the *GigaScience* GigaDB database [37].

## Abbreviations

AGPL: Affero General Public License; CFR: Code of Federal Regulation; CSS: Cascading Style Sheets; DAISY: Data Information System; EDDA: Evidence in Documents, Discovery, and Analytics; EEA: European Economic Area; EU: European Union; FAIR: Findable Accessible Interoperable Reusable; GA4GH: Global Alliance for Genomics and Health; GDPR: General Data Protection Regulation; HGNC: HUGO (Human Genome Organisation) Gene Nomenclature Committee; HTML: Hypertext Markup Language; IT: information technology; PI: principal investigator; UML: Unified Modeling Language.

## Competing Interests

The authors declare that they have no competing interests.

## Funding

This work was (partially) funded by the contribution of the Luxembourg Ministry of Higher Education and Research towards the Luxembourg ELIXIR Node.

## Authors’ Contributions

R.B. designed the DAISY information model, wrote parts of the paper, and supervised the work. P.A. contributed to the information model and software development and wrote parts of the paper. V.G., Y.J., J.L., K.R., and C.T. contributed to the information model and software development and reviewed drafts of the paper. S.M., V.S., and R.S. contributed to the information model and reviewed drafts of the paper.

## Supplementary Material

giz140_GIGA-D-19-00130_Original_Submission

giz140_GIGA-D-19-00130_Revision_1

giz140_GIGA-D-19-00130_Revision_2

giz140_Response_to_Reviewer_Comments_Original_Submission

giz140_Response_to_Reviewer_Comments_Revision_1

giz140_Reviewer_1_Report_Original_Submission Jui-Chu Lin -- 5/31/2019 Reviewed

giz140_Reviewer_2_Report_Original_Submission John Wise, MA -- 8/23/2019 Reviewed
