# Supplementary material for: DAISY: A Data Information System for accountability under the General Data Protection Regulation
Source: Gigascience. 2019 Dec 4;8(12):giz140. doi: 10.1093/gigascience/giz140 (PMC6892452; doi:10.1093/gigascience/giz140)
Supplement: giz140_GIGA-D-19-00130_Original_Submission [file giz140_giga-d-19-00130_original_submission.pdf]

GigaScience

DAISY: a Data Information System for accountability under the General Data Protection Regulation

--Manuscript Draft--

**Manuscript Number:**	GIGA-D-19-00130
**Full Title:**	DAISY: a Data Information System for accountability under the General Data Protection Regulation
**Article Type:**	Technical Note
**Funding Information:**	Université du Luxembourg (Contributions of Ministry of Higher Education and Research towards ELIXIR Luxembourg.) — Dr Reinhard Schneider
**Abstract:**	Background<br><br>The new European legislation on data protection, namely the GDPR, has introduced comprehensive requirements for the documentation about the processing of personal data as well as informing the data subjects of its use. GDPR’s accountability principle requires institutions, projects and data hubs to document their data processings and demonstrate compliance with the GDPR. In response to this requirement, we see the emergence of commercial data mapping tools, and institutions creating GDPR data registers with such tools. One shortcoming of this approach is the genericity of tools, and their process-based model not capturing the project-based, collaborative nature of data processing in biomedical research.<br><br>Findings<br><br>We have developed a software tool to allow research institutions to comply with the accountability requirement and map the sometimes very complex data flows in biomedical research. By analysing the transparency and record keeping obligations of each GDPR principle we observe that our tool effectively meets the accountability requirement.<br><br>Conclusions<br><br>The GDPR is bringing data protection to the center stage in research data management; necessitating dedicated tools, personnel and processes. Our tool, DAISY, is tailored specifically for biomedical research and can help institutions in tackling the documentation challenge brought about by the GDPR. DAISY is made available as a free and open-source tool on Github. DAISY is actively being used at the Luxembourg Centre for Systems Biomedicine and the ELIXIR-Luxembourg data hub.
**Corresponding Author:**	Pinar Alper, Ph.D.<br>University of Luxembourg<br>Esch-sur-Alzette, LUXEMBOURG
**Corresponding Author Secondary Information:**	
**Corresponding Author's Institution:**	University of Luxembourg
**Corresponding Author's Secondary Institution:**	
**First Author:**	Regina Becker, PhD
**First Author Secondary Information:**	
**Order of Authors:**	Regina Becker, PhD
	Pinar Alper, PhD
	Valentin Groues

Powered by Editorial Manager® and ProduXion Manager® from Aries Systems Corporation

	Sandrine Munoz, PhD
	Yohan Jarosz
	Jacek Ledbioda
	Kavita Rege
	Christophe Trefois, PhD
	Venkata Satagopam, PhD
	Reinhard Schneider, PhD
**Order of Authors Secondary Information:**	
**Additional Information:**	
**Question**	**Response**
Are you submitting this manuscript to a special series or article collection?	No
**Experimental design and statistics**<br><br>Full details of the experimental design and statistical methods used should be given in the Methods section, as detailed in our Minimum Standards Reporting Checklist. Information essential to interpreting the data presented should be made available in the figure legends.<br><br>Have you included all the information requested in your manuscript?	No
If not, please give reasons for any omissions below.<br><br>as follow-up to "**Experimental design and statistics**<br><br>Full details of the experimental design and statistical methods used should be given in the Methods section, as detailed in our Minimum Standards Reporting Checklist. Information essential to interpreting the data presented should be made available in the figure legends.<br><br>Have you included all the information requested in your manuscript?<br><br>"	The paper does not include a data supplement or statistical data analysis. Therefore this category is not applicable.

Powered by Editorial Manager® and ProduXion Manager® from Aries Systems Corporation

**Resources**<br><br>A description of all resources used, including antibodies, cell lines, animals and software tools, with enough information to allow them to be uniquely identified, should be included in the Methods section. Authors are strongly encouraged to cite Research Resource Identifiers (RRIDs) for antibodies, model organisms and tools, where possible.<br><br>Have you included the information requested as detailed in our Minimum Standards Reporting Checklist?	Yes
**Availability of data and materials**<br><br>All datasets and code on which the conclusions of the paper rely must be either included in your submission or deposited in publicly available repositories (where available and ethically appropriate), referencing such data using a unique identifier in the references and in the "Availability of Data and Materials" section of your manuscript.<br><br>Have you have met the above requirement as detailed in our Minimum Standards Reporting Checklist?	Yes

Paper compiled with GIGASCIENCE latex template
Click here to download Manuscript bdvqkmkxfxdysvyyjbbmddxbgbwyqbxd.pdf

GigaScience, 2019, 1–9

doi: xx.xxxx/xxxx
Manuscript in Preparation
Technical Note

TECHNICAL NOTE

DAISY: a Data Information System for accountability under the General Data Protection Regulation

Regina Becker[1], Pinar Alper[1], Valentin Groues[1], Sandrine Munoz[1], Yohan Jarosz[1], Jacek Lebioda[1], Kavita Rege[1], Christophe Trefois[1], Venkata Satagopam[1] and Reinhard Schneider[1]

[1]Luxembourg Centre for Systems Biomedicine, University of Luxembourg

name.surname@uni.lu

Abstract

Background
The new European legislation on data protection, namely the *GDPR*, has introduced comprehensive requirements for the documentation about the processing of personal data as well as informing the data subjects of its use. GDPR's *accountability* principle requires institutions, projects and data hubs to document their data processings and demonstrate compliance with the GDPR. In response to this requirement, we see the emergence of commercial *data mapping* tools, and institutions creating GDPR data registers with such tools. One shortcoming of this approach is the genericity of tools, and their process-based model not capturing the project-based, collaborative nature of data processing in biomedical research.
Findings
We have developed a software tool to allow research institutions to comply with the accountability requirement and map the sometimes very complex data flows in biomedical research. By analysing the transparency and record keeping obligations of each GDPR principle we observe that our tool effectively meets the accountability requirement.
Conclusions
The GDPR is bringing data protection to the center stage in research data management; necessitating dedicated tools, personnel and processes. Our tool, DAISY, is tailored specifically for biomedical research and can help institutions in tackling the documentation challenge brought about by the GDPR. DAISY is made available as a free and open-source tool on Github. DAISY is actively being used at the Luxembourg Centre for Systems Biomedicine and the ELIXIR-Luxembourg data hub.

Key words: GDPR; Accountability; Data mapping

Background and Motivation

The General Data Protection Regulation (GDPR) [1] is a European Union (EU) regulation on data protection that is a directly applicable law in all EU member states since 25th May 2018. In addition, the GDPR has been incorporated into the European Economic Area (EEA) agreement and is therefore applicable to the member states of the European Free Trade Association (EFTA) as of 6th July 2018 [2]. In practice, however, the reach of the GDPR spans the whole world; where services or goods are being offered to citizens in the EU, the corresponding institution needs to comply with the GDPR, independent of where it is located. Furthermore, there is an obligation for data controllers in the EU to ensure that recipients outside the EU adhere to GDPR equivalent data protection standards wherever possible. Due to its global impact, the GDPR affects the international research community and it has consequences for the way research data will be handled in the future [3].

One important new principle introduced by the GDPR in Article 5 is "accountability". Accountability requires the demon-

Compiled on: April 17, 2019.
Draft manuscript prepared by the author.

Key Points

- Data collected from human subjects for research fall under the scope of GDPR.
- The accountability principle of GDPR requires institutions, projects or data hubs to document their compliance with the principles of the GDPR.
- We have developed an open-source software tool to address all relevant points of the GDPR and map them to the data processing within collaborative biomedical research.

stration, and thus documentation, of the compliance with all data protection principles: lawfulness, fairness, transparency, purpose limitation, data minimisation, accuracy, storage limitation, integrity and confidentiality. While these principles were already the basis of the previous data protection directive [4], **the necessity to record compliance is new**. GDPR Article 6 outlines six possible "lawful bases" for processing personal data. Research institutions need to document under which basis their processings fall. For particular bases, such as "legitimate interest", institutions need to perform assessments justifying this legal ground with "balancing tests" or "data protection impact assessments" [5][6]. Article 30 of the GDPR outlines that institutions should record "data processings" and the records should at a minimum contain the following:

- Stakeholders, their roles (controller, processor) and contact information;
- Purposes of data processing;
- Categorical descriptions of data subjects and the data held on subjects;
- Categorical descriptions of recipients of data, particularly those in non-EU countries and international organisations;
- Logs of data transfers to identified recipients and a description of safeguards put in place;
- Where possible, time limits for keeping different categories of personal data;
- Where possible, descriptions of organisational and technical data protection measures.

GDPR requires that records of data processing should be auditable by supervisory authorities. Some countries such as Slovakia or Spain can even impose fines if these records are not in a suitable form for auditing [7] [8].

In biomedical research, data collected from human subjects and their biological specimens fall under the GDPR when such data is not anonymous. While GDPR's accountability principle is often mentioned in biomedical literature, we see that only few publications discuss its implications, and then only briefly [9] [10] [11] .

In response to the documentation requirement, we see the emergence of commercial "data mapping" tools, and academic institutions creating GDPR data registers with such tools [12] [13]. We observe that emerging approaches typically have three particular shortcomings for documenting the processing of personal data in biomedical research.

i. Documentation is typically done on a per process basis. An institution's activities, such as personnel administration or teaching, are recorded in terms of the processes involved, such as staff recruitment or student registration etc. When it comes to documenting research, however, the process-based approach falls short. Research is undertaken via projects, and each project represents a distinct report-worthy configuration in terms of, what are the GDPR roles of research collaborators, what types of data are used, their de-identification status and for how long the respective data can be kept.

ii. Categorical descriptions of data subjects and data recipients do not provide sufficient transparency when handling data subjects' requests for information. For example, Article 30 requires only the listing of categories of recipients with whom personal data has been shared, whereas Article 15 states that the data subject has the right to know the individual recipients or, if this is not possible, the categories of recipients. In addition, the source of the data needs to be provided on request as well as criteria for the retention of the data if no specific time limit is given.

iii. A distinct characteristic of personal data processing in biomedical research is the separation of the collection of data from its continued processing. It is common to collect data from a cohort with a broad consent, as - due to the nature of research - possible uses cannot always be fully enumerated at the time of collection. In such cases, projects that collect, process or further process data may be operating under different legal grounds (consent, public interest, legitimate interest), which need to be recorded to demonstrate the compliance with Article 6 of the GDPR.

In this paper we describe a software tool, named the Data Information System (DAISY), which can be used to meet the data documentation requirement that the GDPR imposes on biomedical research. The remainder of the paper is organised as follows. We first introduce our method for data documentation and the target users of DAISY. Afterwards we discuss DAISY's information model in detail and typical ways of inquiring this model. In findings, we discuss how DAISY supports compliance with GDPR's accountability principle. We conclude the paper with final remarks and pointers to DAISY source code.

Method

DAISY has been developed to serve as a registry for research data processing that supports compliance with the GDPR and can serve for both auditing by the supervisory authorities and fulfilment of requests by data subjects.

During a GDPR audit, institutions need to provide information about what data they have, the lawful basis for data processing, as well as an appropriate record of the processing itself. In order to satisfy these requirements, one needs to pull together information from both human and machine sources (see Figure 1).

Research Principal Investigators (PIs) have expert knowledge about the projects, the human bio-samples and data used as well as the collaborators that are the source or recipient of such data. The individual researchers know where and how data is held and processed. The legal department (Legal Team) establishes and maintains various kinds of contracts with collaborators and tracks the GDPR compatibility of those contracts. The information technology (IT) and systems administration personnel (IT Team) has an overview of what storage and transfer mechanisms are available for researchers, e.g. the submission portal where external collaborators can upload their data

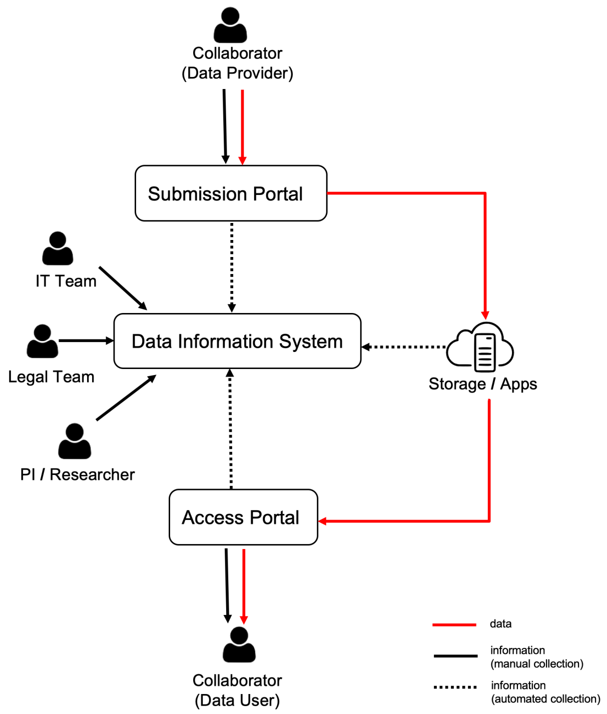

Figure 1. DAISY Target users and information flow.

or the access portal where they can download or process data onsite. The IT Team is also knowledgeable about the technical protection measures such as access control and encryption. Information from these stakeholders is typically distributed and in an unstructured form (e.g. email communication, documents, wiki/intranet).

In addition, the IT infrastructure and tools for data transfers, storage and management should generate evidential information that can be used to demonstrate how data processing takes place in practice. Examples are timestamped logs of user actions on data, granted or rejected accesses, or access permissions for a resource.

With DAISY, we initially focused on the manual collection of information from people. The next phase of development will focus on plugins that can automatically harvest evidential data processing logs from IT infrastructure. In the following section, we outline the DAISY information model, and the possible inquiries that can be made over this model.

DAISY Information Model

At its core, DAISY is focused on recording research studies, the use of personal data of study participants, the sources of data and the legal grounds for processing data. DAISY's key entities are summarised in Table 1 for quick reference, the detailed model is given as a UML class diagram [14] in Figure 2.

Project represents a research activity occurring over a certain period of time. **Projects** can be organised in hierarchies; the long-term research programme of a laboratory can be recorded as an umbrella project and individual researchers' work can be recorded as its sub-projects. DAISY allows **Projects** to be tagged with controlled vocabulary terms to denote study features, as well as the study focus, such as diseases, genes or phenotype attributes. The terms build, where possible, on standards, by default DAISY incorporates EDDA Study Designs Taxonomy [15], Human Phenotype Ontology (HPO) [16], Human Disease Ontology (HDO) [17] and Human Gene Nomenclature Symbols (HGNC) [18]. These can be replaced with ontologies of choice for a particular DAISY deployment.

Projects have one or more attached **Documents**. **Documents** are typically produced during the ethical, legal and administrative procedures followed during Project implementation. **Documents** can either be stored in DAISY, or they can also be linked to it when they are archived in external document management systems. DAISY uses flags to track a project's status on meeting national and institutional ethics requirements. In case of an absence of approvals, the user is expected to provide justifications. As per GDPR Article 22, it is necessary to track activities involving automated profiling of human subjects. An example of profiling in biomedical research is the calculation of disease ratings or scores from clinical attributes. DAISY flags such projects to enable reporting during audits. All stakeholders of a project including Principal Investigators, research and administrative personnel can be recorded in DAISY. These could either be **Contacts** in **Partner** institutes or local personnel, which are recorded as Users in DAISY. The primary responsible person for the GDPR documentation of human data used in research is the Principal Investigator, and these users are marked as local custodians of projects and data.

Contract is another key category in DAISY utilised for data provenance tracking of incoming or outgoing data. A **Contract** is a legal agreement with one or more signing **Partners**, and it is typically associated with one or more legal **Documents**.

A **Project's** data is recorded as a **Dataset** containing one or more **Data Declarations**. A **Dataset** is a physical or logical unit of data, which is typically treated as a resource (e.g. a folder of files, a database or a lab book) with an associated storage endpoint and access control policy. Meanwhile, a **Data Declaration** represents the smallest unit of data that is traceable to a distinct data source. Data can be declared in the following three ways, depending on its source:

- Data that is being obtained from a **Partner** (collaborator or repository) for the purposes of a project. These declarations will point to the source **Contract** with the **Partner**.
- Data that has been obtained in an earlier project, and is being re-used in a follow-on project. These declarations will point to an existing **Data Declaration** as their source.
- Data that has not been obtained from a collaboration **Partner** or an earlier **Project**, such as data downloaded from community repositories. These declarations will have a source description in free text.

In addition to the above information on the data's source, DAISY facilitates the maintenance of a list of **Cohorts**, and links **Data Declarations** with source **Cohorts**. A **Cohort** represents a study that collects data and/or biosamples from a group of participants (e.g. longitudinal case-control or family studies). With **Cohort** annotations on **Data Declarations**, one can refer to all data that was generated around a group of data subjects. This is especially useful for the effective handling of a subject's requests. For instance, when a subject withdraws consent or exercises his/her right of access, the affected projects and data can be easily identified using **Cohort** annotations.

DAISY tracks data **Use Restrictions** per **Data Declaration**. Within one cohort, subject consents and the resulting data processing permissions given by each subject may differ. We model this using a consent status flag on **Data Declarations**. This field denotes whether subject consents are heterogeneous or homogeneous. **Data Declarations** with homogeneous consent would have a single **Use Restriction** configuration, meanwhile those with heterogeneous consent have multiple **Use Restriction** configurations. The **Use Restriction** entity in DAISY models a restriction using a combination of the machine readable "consent code" established by the Global Alliance for Ge-

**Project** is a time-limited research activity with associated documentation on the ethical, legal and administrative procedures carrying out its implementation.
**Partner** is a research collaborator that is the source and/or recipient of human data. Partners are also legal entities with whom contracts are signed. Clinical entities that run longitudinal cohorts, research institutes, or data hubs are examples of Partners.
**Contract** is a legal agreement with one or more partners. Contracts are established by one or more mutually-signed documents, data sharing agreements, consortium agreements, material transfer agreements are examples of contracts.
**Dataset** is a physical/logical unit of data, which is typically treated as a resource with an associated location and access control policy.
**Data Declaration** is a sub-unit of data, which is traceable to a particular source, which could be the provider partner and source cohort, or another data declaration.
**Cohort** is a study that collects data and/or bio-samples from a group of participants (e.g. longitudinal case-control or family studies). A cohort is linked to the creation of data and is considered its ultimate source.

Table 1. Overview of key DAISY entities

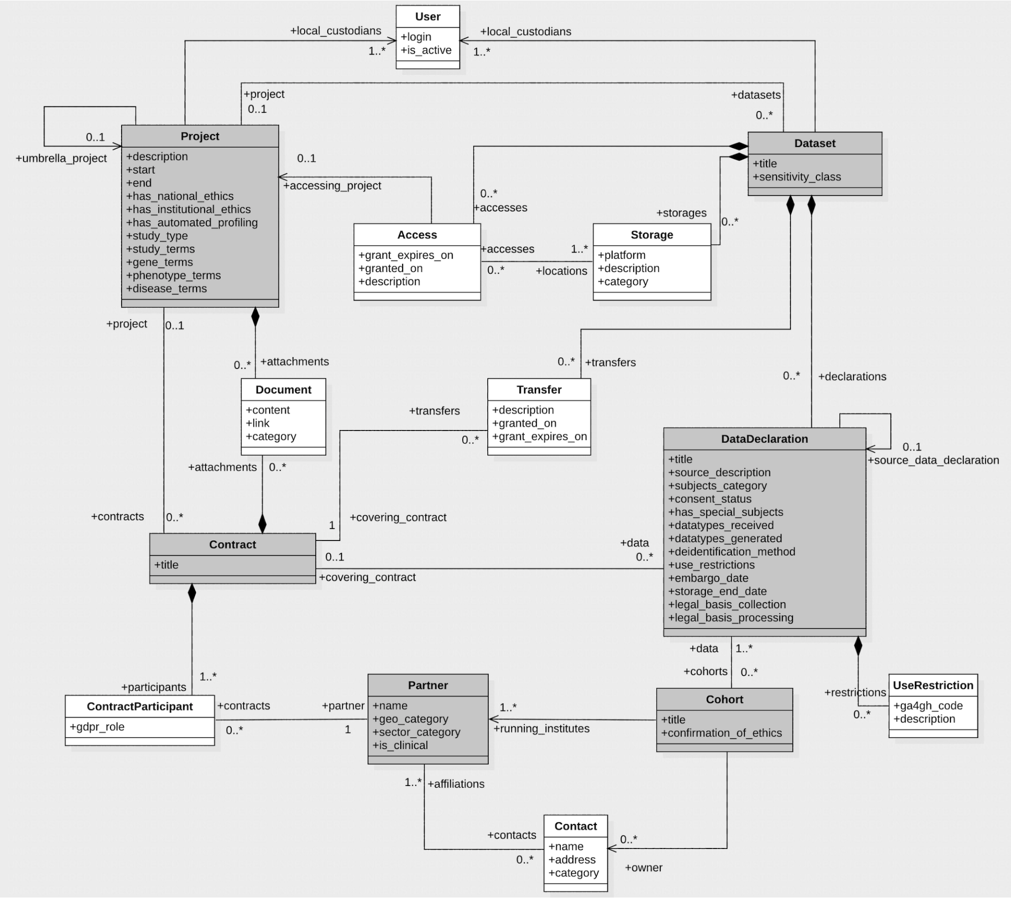

Figure 2. DAISY Information model.

nomics and Health (GA4GH) [19] and free-text descriptions. Examples of consent codes are project or disease or research area specific restrictions on data use. DAISY actively monitors time-dependent restrictions such as embargo periods or storage durations, and sends date expiry notifications to responsible users. GDPR puts special emphasis on data of "special subjects" such as minors or those unable to give consent. DAISY also allows the flagging of special subjects and documenting subject categories (cases, controls, etc.). For each **Data Declaration**, DAISY requires the recording of data types received e.g. biosamples received, or metabolomics and whole exome data generated.

DAISY records "data processing", such as data access, storage and thrid-party transfer, at the level of **Datasets**. For **Storage**, DAISY allows the definition of a set of platforms on which data could possibly be stored. These can be applications, relational databases or file systems. This is relevant for the technical safeguards that go along with the respective platform. **Storage** is then defined in terms of a platform and a textual description (e.g. actual link to the data). The policy on how local stakeholders, e.g. researchers or IT personnel, can access a dataset is captured with the **Access** entity. **Access** definitions refer to **Storage** locations, together with a free-text description of the access control policy and its validity period. **Transfer** of a dataset to external parties (EU/non-EU) is recorded with its timestamp, the recipient **Partner**, the **Contract** that establishes the legal basis of **Transfer** and a free-text description of safeguards taken for the transfer.

Human data in research can be of varying sensitivity and correspondingly varying protective measures are needed. For instance, whole genome data, when accompanied with disease phenotypes is considered highly sensitive and should be kept encrypted at rest [20]. During a GDPR audit, the handling of such data can be a particular focus. In order to support such in-

quiries, DAISY allows for defining data sensitivity classes that can flag data of higher sensitivity requiring additional safeguards and the tagging of **Datasets** with those classes.

Inquiring DAISY

A sample depiction of relations among DAISY entities is given in Figure 3. DAISY information space is commonly explored from three perspectives, based on **Datasets**, **Projects** and **Contracts**.

i. **Datasets** can be located on host storage platforms based on **Cohorts** covered or the types of data contained. From here the user can navigate to the **Projects** that (re)use this data as well as its sources and the **Contracts** and **Documents** ensuring its data's legal and ethical processing.
ii. **Projects** are indexed with their responsible Principal Investigators, and are classified with respect to subject areas and study methods. From a **Project**, one can navigate to **Datasets** that are used in the scope of this project, including those made accessible from other **Projects**. **Projects** also link to **Contracts** and associated **Partners** that have sent or received data.
iii. **Partners** are indexed according to their geographic location, sector and activity e.g. as a healthcare institution. It is possible to get an overview of **Contracts** signed with designated **Partners**. From one **Contract**, e.g. a consortium agreement, one can navigate to the encompassing **Project** and then to other **Contracts** such as material transfer or data sharing agreements.

All inquiries start by locating records of interest and navigating to other related records. DAISY provides faceted search pages for all major entities and allows the traversal of links between entities. A screenshot of the Partner search interface given in Figure 4.

Findings

At its core, DAISY needs to provide all the information required to fulfil the transparency and record keeping obligations of Articles 15 (right of access) and 30 (records of processing activities). However, for accountability, by following Article 5, further documentation is needed as described below. This combined information fulfils the requirements for accountability in the following way.

Lawfulness

The legal basis for the processing is a mandatory field in DAISY. Additional entries enable either a reference to be made to external files or the upload of files that can further corroborate the chosen legal basis. In the case of legitimate interest, the balancing exercise will be needed. For consent as legal basis, the consent template is relevant. When the public interest basis is used, the reference to the respective law required following the GDPR Article 6(3) should be provided. The legal basis is not a mandatory element of the record keeping in Article 30, however, it is crucial to document it to properly comply with the GDPR.

Fairness

Documenting the fairness is a challenging undertaking. As an example, the UK Data Protection Act [21] states in Article 19(2) that the research must not cause substantial damage or substantial distress to a data subject. Within DAISY, the fairness principle for a clinical study is covered by the reference to, or the upload of, the participant information sheet and the corresponding consent form. These documents demonstrate the agreement of the study participants with the intended research. In addition, the ethics approval documents give an independent assessment that the purposes of the research do not override the interests of the study participants in an unfair way. The approach to prove fairness through an ethics approval is in some countries even part of the legislation, e.g. in the UK [21] or in Sweden [22].
Where data is imported from repositories and collaborators, the confirmation of the existence, and the coverage of, the ethics approval is obtained as part of the data sharing agreement. This document also determines the purposes for which the data can be used in compliance with the fairness principle. In this case, no consent forms are needed and the ethics approval documents uploaded in DAISY will cover only the research project using the data.

Transparency

The fields in DAISY cover all information requirements about the processing towards the data subject according to Articles 13, 14 and 15. The purpose of the field"project description is to provide a lay summary that is understandable for the data subject. In addition, the record keeping following Article 30 is addressed within DAISY when the information is specific for the particular project. The more generic information on the technical and organisational security measures has to be documented outside DAISY (see below under confidentiality).

Purpose limitation

The overall purpose of processing data for research is inherent for all research projects using the data. However, often the data has been collected for more specific reasons. Here, the scope of the consent of the study participants is the guiding principle, even if the chosen legal basis is not consent but e.g. public interest or legitimate interest. Data collection in the medical research context requires a consent, wherever possible, from the ethics point of view, independent of data protection law [23][24][25][26]. Therefore, each dataset has a purpose limitation field that follows the machine readable GA4GH codification [19]. This enables automated consent management to be done at the study level. In addition, the Consent Status field specifies if the obtained consent has been homogeneous or heterogeneous. As the GDPR requires the consent to be given for each purpose individually [27], studies often offer a selective option to consent to the specific study in which context the data is collected as well as additional future research projects, thus giving a broad consent for the data use in research. DAISY has a field to store the column(s) in the tabular clinical information that specifies the different consent options. With this, information about the position in the clinical data of the participants' consent can be found and used for subject level consent management. This enables an implementation of the purpose limitation at the level of individual subjects.

Data minimisation

Data minimisation needs to be implemented on two levels, the administrative data within DAISY and the research data administered by DAISY. On the administrative side, DAISY foresees a role definition of different stakeholders with corresponding access rights (profile based). To allow data minimisation of research data, DAISY lists the different types of data available in the datasets. This list gives input for the data analysis plan that should be uploaded as part of the project description and thus can provide the basis for a selective access provision.

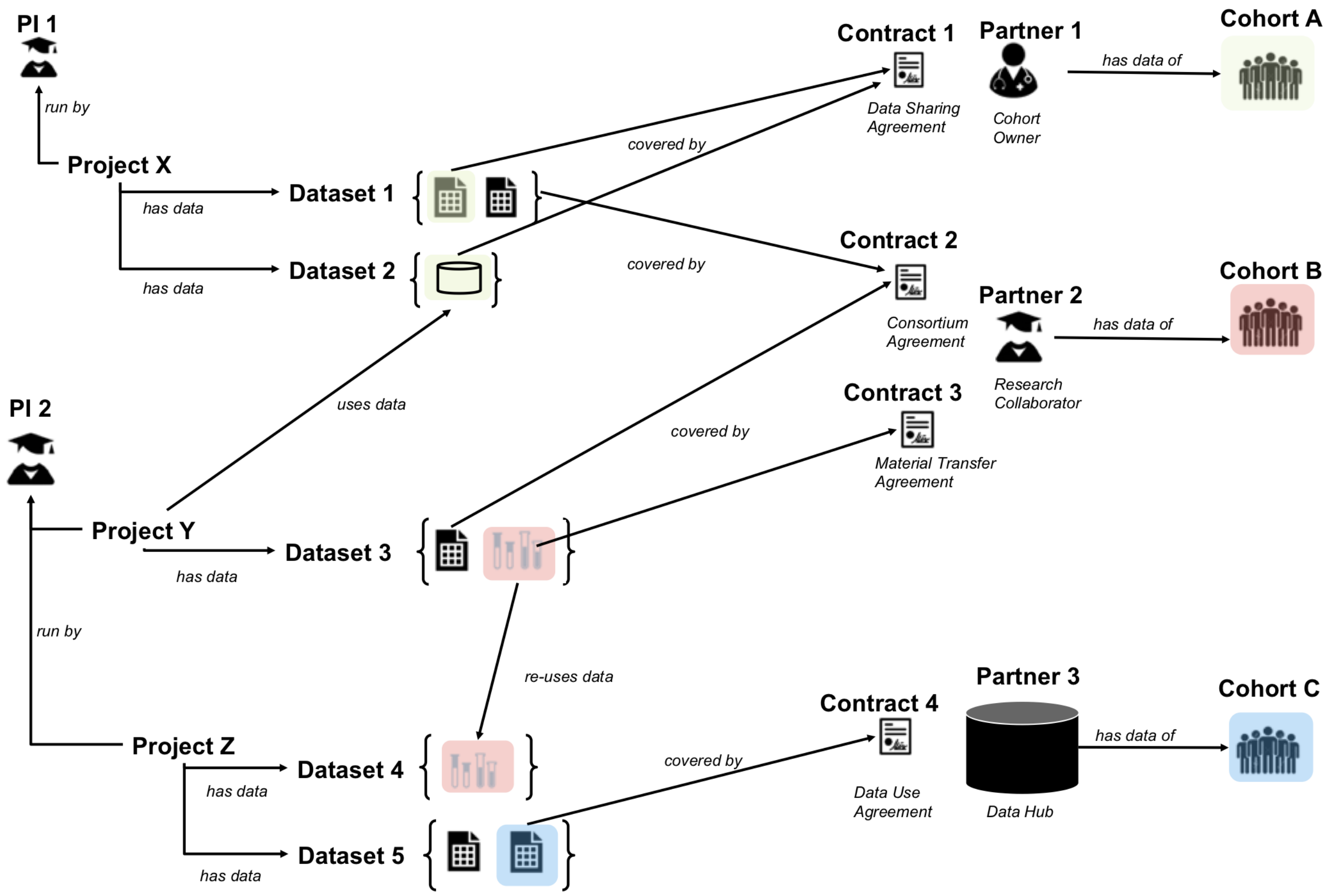

Figure 3. Example DAISY records and the relationships between them. **Projects** and **Datasets** create a matrix of associations. A **Project** may involve several **Datasets** with (sub)**DataDeclarations** coming from different source **Cohorts** and covered by different **Contracts** with various **Partners**, which may have been signed at different times. Additionally, data/biosamples obtained for one **Project** may be re-used in another **Project** (biosamples in Dataset 3 of Project Y are re-used in Project Z Dataset 4). Furthermore, a **Project** may access data of other projects (Project Y and Dataset 2). Typically, **Cohort** data is collected for an open number of projects. Therefore, biosamples and data of one cohort may end up in several datasets within an institution; we denote this with colours, data from a specific **Cohort** is tainted with the colour of that **Cohort**. Also, note that researchers may generate different data types using biosamples/data obtained from a **Cohort** e.g. generate genomics data from samples. DAISY ensures that **Cohort** annotations are propagated to newly generated (derivative) data.

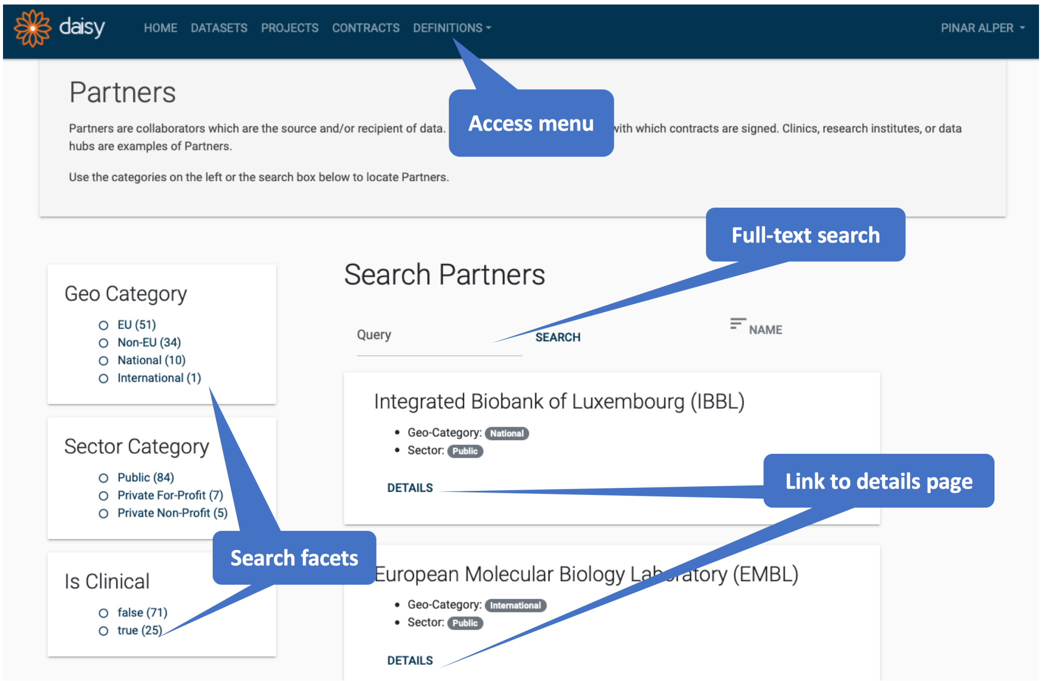

Figure 4. Screenshot of Partner search page in DAISY.

Accuracy
Information related to the data accuracy for the research data can be provided by linking to a metadata file or a management system that contains additional information on the data collected and standard operating procedures (SOPs) used. The metadata link can be found in the source project that describes the study or origin that led to the creation of the dataset. The accuracy of the administrative data of DAISY is to be achieved by the different access rights of the stakeholders editing DAISY. Some information, e.g. relevant legal data, can only be edited or changed by a legal administrator to avoid erroneous entries. In addition, record updates in DAISY are logged; the timestamp of updates and the user performing the operation are recorded.

Storage limitation
The retention time for a dataset is an entry in DAISY. An active alerting system informs the responsible PI as well as the legal team and the data steward before the foreseen storage duration comes to an end.

Integrity
The data integrity can be followed up through the creation of the checksum [28] value that stays with the dataset during its lifetime from the ingestion into the IT infrastructure until its deletion. While the checksum is usually kept together with the respective dataset, it can also be saved within DAISY to keep track of it. The checksum value can be repeatedly tested to avoid silent corruption of the data and can be transmitted with each data transfer to ensure that no data is corrupted during transit.

Confidentiality
Both data controller and processor are obliged to implement appropriate technical and organisational measures to ensure the privacy of the data subjects (GDPR Articles 24, 25, 26 and 32). The organisational and technical security measures need to be documented following Article 30 of the GDPR. Rather than documenting all security measures directly in DAISY, a link to such external documentation is foreseen. Likewise, part of each project characterisation in DAISY is a link to a data protection impact assessment (DPIA) that is performed externally.

National differences
Ultimately, full compliance with data protection law may be subject to national or regional differences depending on the interpretation by the data protection authorities and the GDPR implementations in the different countries through local laws. Here, not only data protection law may be relevant but also subsequent sector laws regulating areas like healthcare, biobanking or research where additional requirements can be defined. Therefore, it is necessary for all data controllers to be aware of the relevant legislation in their countries. Data protection officers in the institutions can help in this quest. Where the requirements are not clear, the relevant data protection authority can be requested to provide clarification.

Conclusion

DAISY can assist research institutions with a guidance on their documentation obligations and can serve as a knowledge base about the data existing in the institution. DAISY not only supports documentation towards accountability but also helps to comply with the responsibility principle as it warns, for instance, about the expiry of data retention times. It also gives indications about what further research is compatible with the existing consent by matching information on potential future projects with the consent codes stored for the data.

While the extensive documentation required may seem excessive, the GDPR actually puts in place what has already been postulated many years ago for health databases from an ethical point of view, such as the World Medical Association (WMA) declaration on ethical considerations on health databases and biobanks [29]. With audits and the corresponding fines for non-compliance, the GDPR now enforces such ethical principles.

As the documentation obligations described above are a new requirement under the GDPR, not many research institutions are ready for this administrative challenge. A particular hurdle is that for all personal data acquired prior to 25 May 2018, the same documentation is needed if the data are still being processed. This includes the mere storage of personal data. Substantial catching up will be needed.

The GDPR is leading to new positions in the research environment: as per Article 37, all public institutions as well as institutions processing sensitive data on a large scale are required to have a data protection officer. The administrative and documentation efforts around the data processing may also lead to increased employment of data stewards who ensure the proper ingestion and registration of data in systems such as DAISY. Such developments driven by data protection law may, however, also improve the quality of the data as the support given by data stewards can also lead to better documentation of scientific metadata and better structuring of databases. Data protection will ultimately become part of a research institution strategy in their efforts to make data FAIR: findable, accessible, interoperable and reusable [30]. DAISY was developed as a tool to support this work.

Availability of source code and requirements

DAISY has been developed as a Python web application. Bootstrap and jQuery are used for the front-end (HTML, CSS, Javascript). PostgreSQL is used as the database backend, and SolR is used as the full-text search engine. The DAISY source code is available online [31] with a GNU Affero General Public License (AGPL). DAISY installation has primarily been tested on Linux CentOS 7 servers; DAISY also has a simpler Docker-based deployment, which can be used for demo deployments and software evaluation. Instructions for both deployments can be found in our source repository [31].

DAISY has interfaces to allow the upload of CSV or Excel files to import relevant data from other information systems or following the independent collection of information. Also, a planned feature is to have exports of data provenance information in DAISY. Such exports can support requests for information during data protection audits or when responding to study participants' requests.

Declarations

List of abbreviations

AGPL – Affero General Public License
CSS – Cascading Style Sheets
DAISY – Data Information System
EDDA – Evidence in Documents, Discovery, and Analytics
EEA – European Economic Area
EFTA – European Free Trade Association
EU – European Union
FAIR – Findable Accessible Interoperable Reusable
GA4GH – Global Alliance for Genomics and Health
GDPR – General Data Protection Regulation
HDO – Human Disease Ontology

HGNC – HUGO (Human Genome Organisation) Gene Nomenclature Committee
HPO – Human Phenotype Ontology
HTML – Hypertext Markup Language
UML – Unified Modeling Language
WMA – World Medical Association

Ethical Approval

Not applicable.

Consent for publication

Not applicable.

Competing Interests

The authors declare that they have no competing interests.

Funding

This work was (partially) funded by the contribution of the Luxembourg Ministry of Higher Education and Research towards the Luxembourg ELIXIR Node.

Authors' Contributions

Regina Becker designed the DAISY information model, wrote parts of the paper and supervised the work. Pinar Alper contributed to the information model and software development and wrote parts of the paper. Valentin Grouès, Yohan Jarosz, Jacek Lebioda, Kavita Rege and Christophe Trefois contributed to the information model and software development and reviewed drafts of the paper. Sandrine Munoz, Venkata Satagopam and Reinhard Schneider contributed to the information model and reviewed drafts of the paper.

References

1. Regulation (EU) 2016/679 of the European Parliament and of the Council of 27 April 2016 on the protection of natural persons with regard to the processing of personal data and on the free movement of such data, and repealing Directive 95/46/EC (General Data Protection Regulation). EUR-Lex - 32016R0679 - EN. http://data.europa.eu/eli/reg/2016/679/oj
2. Decision of the EEA Joint Committee No 154/2018 of 6 July 2018 amending Annex XI and Protocol 37 to the EEA Agreement [2018/1022]. EUR-Lex - 22018D1022 - EN. http://data.europa.eu/eli/dec/2018/1022/oj
3. Shabani M, Borry P. Rules for processing genetic data for research purposes in view of the new EU General Data Protection Regulation. European Journal of Human Genetics. 2018 Feb; 26(2):149-156. doi: 10.1038/s41431-017-0045-7.
4. Directive 95/46/EC of the European Parliament and of the Council of 24 October 1995 on the protection of individuals with regard to the processing of personal data and on the free movement of such data. https://eur-lex.europa.eu/legal-content/EN/TXT/?uri=celex:31995L0046
5. UK Information Commissioner's Office. Guide to the General Data Protection Regulation: Legitimate Interests. https://ico.org.uk/for-organisations/guide-to-the-general-data-protection-regulation-gdpr/lawful-basis-for-processing/legitimate-interests/
6. EC Data Protection Working Party. Article 29 Working Party Opinion 06/2014 on the notion of legitimate interests of the data controller under Article 7 of Directive 95/46/EC. https://ec.europa.eu/justice/article-29/press-material/public-consultation/notion-legitimate-interests/files/20141126_overview_relating_to_consultation_on_opinion_legitimate_interest_.pdf
7. National Council of the Slovak Republic. Act No. 122/2013 Coll. on Protection of Personal Data and on Changing and Amending of other acts, resulting from amendments and additions executed by the Act. No. 84/2014 Coll. https://www.dataprotection.gov.sk/uoou/sites/default/files/kcfinder/files/Act_122-2013_84-2014_en.pdf
8. Spain: The Organic Law 3/2018 of 5 December on the Protection of Personal Data and the Guarantee of Digital Rights Art 74(12). https://www.boe.es/boe/dias/2018/12/06/pdfs/BOE-A-2018-16673.pdf
9. The European General Data Protection Regulation: challenges and considerations for iPSC researchers and biobanks. Regenerative Medicine. 2017 Sep;12(6):693-703. doi: 10.2217/rme-2017-0068.
10. Chassang G. The impact of the EU general data protection regulation on scientific research. Ecancermedicalscience vol. 11 709. 3 Jan. 2017. doi:10.3332/ecancer.2017.709.
11. Dove ES. The EU General Data Protection Regulation: Implications for international scientific research in the digital era. Journal of Law, Medicine and Ethics, Vol 46 (2018).
12. Vigilant Software Dataflow Mapping Tool. https://www.vigilantsoftware.co.uk/topic/data-flow-mapping-tool
13. OneTrust Data Mapping Automation. https://www.onetrust.com/products/data-mapping
14. Rumbaugh JE, Jacobson I, Booch G. Unified Modeling Language Reference Manual, the (2nd Edition 2004). Pearson Higher Education. ISBN:0321245628.
15. Bekhuis T, Tseytlin E. Evidence in Documents, Discovery, and Analytics (EDDA) Study Designs Taxonomy (2016, version 2.0). 10.13140/RG.2.1.3769.2406/1.
16. Köhler S, Doelken SC, et al. The Human Phenotype Ontology project: linking molecular biology and disease through phenotype data. Nucleic Acids Res. 2014;42:D966?74. doi: 10.1093/nar/gkt1026.
17. Schriml LM, Mitraka E, et al. Human Disease Ontology 2018 update: classification, content and workflow expansion, Nucleic Acids Research, Volume 47, Issue D1, 8 January 2019, Pages D955?D962, https://doi.org/10.1093/nar/gky1032
18. Braschi B, Denny P, et al. Genenames.org: the HGNC and VGNC resources in 2019. Nucleic acids research, 47(D1), D786-D792.
19. Dyke SOM, Philippakis AA, et al. Consent Codes: Upholding Standard Data Use Conditions.2016 PLOS Genetics 12(1): e1005772. https://doi.org/10.1371/journal.pgen.1005772
20. Tang H, Jiang X, Wang X, et al. Protecting genomic data analytics in the cloud: state of the art and opportunities. BMC Med Genomics. 2016 Oct 13;9(1):63. doi: 10.1186/s12920-016-0224-3.
21. UK Data Protection Act 2018 http://www.legislation.gov.uk/ukpga/2018/12/pdfs/ukpga_20180012_en.pdf
22. Sweden, SFS 2003:460. SFS 2003:460 (2003). Law on ethics testing of human-related research. Stockholm Social Department. https://www.riksdagen.se/sv/dokument-lagar/dokument/svensk-forfattningssamling/lag-2003460-om-etikprovning-av-forskning-som_sfs-2003-460
23. World Medical Association. Declaration of Helsinki ethical principles for medical research involving human subjects (2013). JAMA: 310(20), 2191-2194, doi:

10.1001/jama.2013.281053.
24. The NuGO Bioethics Guidelines on Human Studies. Oslo, September 17th, 2007 http://nugo.dife.de/bot/index.php
25. European Society of Human Genetics PPPC. Data storage and DNA banking for biomedical research: technical, social and ethical issues. European Journal of Human Genetics. 2003 Dec;11(12):906-8. doi: 10.1038/sj.ejhg.5201107
26. Van Delden JJM, Van der Graaf R. Revised CIOMS International Ethical Guidelines for Health-Related Research Involving Humans. JAMA. 2017;317(2):135?136. doi:10.1001/jama.2016.18977.
27. EC Data Protection Working Party. Article 29 Working Party Guidelines on consent under Regulation 2016/679. Adopted on 28 November 2017. https://ec.europa.eu/newsroom/article29/item-detail.cfm?item_id=623051
28. Smoak CG, Widel M, Truong S. The use of checksums to ensure data integrity in the healthcare industry. Pharmaceutical Programming 5(1/2):38-41, December 2012.
29. 67th World Medical Association General Assembly, Taipei, Taiwan. WMA declaration of Taipei on ethical considerations regarding health databases and biobanks. As revised on October 2016 https://www.wma.net/policies-post/wma-declaration-of-taipei-on-ethical-considerations/regarding-health-databases-and-biobanks/
30. Wilkinson MD, Dumontier M, et al. The FAIR Guiding Principles for scientific data management and stewardship. Nature Scientific Data 2016;3. doi: 10.1038/sdata.2016.18.
31. ELIXIR-Luxembourg Github Repository. https://github.com/elixir-luxembourg/daisy

Personal Cover Click here to download Personal Cover CoverLetter.pdf

17 April 2019

To: GIGASCIENCE Editorial Board

Dear Editors,

The General Data Protection Regulation (GDPR) has come into force across all EU countries in 25 May 2018. GDPR significantly impacts all research collecting and using human and bio-samples/data. The biomedical research community is now going through a period of "GDPR interpretation", eliciting how the legislation affects their activities and what "GDPR Compliance" means. The new principles of "responsibility" and "accountability" lead to new requirements in documentation. Institutions are not only expected to protect Human bio-samples and data, they also need to document their compliance.

In a nutshell, GDPR requires researchers to perform data bookkeeping, whenever working with human data/bio-samples. As the obligations from the GDPR are imposed by law, they need to be incorporated into the research process.

In this paper we describe how we turned these new obligations into a software tool that allows biomedical research institutions to comply with GDPR obligations and map the sometimes very complex data flows stemming from collaborative research.

We believe the paper is well suited to GIGASCIENCE, as our tool is open-source and freely available for the use of biomedical research community. With this Technical Note we're aiming to increase awareness about some of GDPR's requirements and how our tool can be used to address them.

Regards

Regina Becker, Pinar Alper, Valentin Grouès, Sandrine Munoz, Yohan Jarosz, Jacek Lebioda, Kavita Rege, Christophe Trefois, Venkata Satagopam, Reinhard Schneider

Luxembourg Centre for Systems Biomedicine,
University of Luxembourg,
Esch-sur-Alzette L-4362,
Luxembourg
