# Supplementary material for: DAISY: A Data Information System for accountability under the General Data Protection Regulation
Source: Gigascience. 2019 Dec 4;8(12):giz140. doi: 10.1093/gigascience/giz140 (PMC6892452; doi:10.1093/gigascience/giz140)
Supplement: giz140_Response_to_Reviewer_Comments_Original_Submission [file giz140_response_to_reviewer_comments_original_submission.pdf]

Author's Response To Reviewer Comments

Close

DAISY: a Data Information System for accountability under the General Data Protection Regulation (Revision submitted on
September 2019)

We would like to that reviewers for their constructive comments. We have revised the paper along those comments. Our individual responses can be found below.

Regards
Authors

Responses:

Reviewer #1:

1. For that the research topic specifically highlight on "accountability," it becomes necessary for the author to explain in th
"Background and Motivation" concerning the reason to include Art. 6 and Art. 30 substantially, so that the readers may be
catch the points concerning the correlation among different articles.

Response:
We added the following sentence to the Background and Motivation section.

“The documentation of the legitimacy of the processing including supporting
information as well as of the processing are important requirements under the GDPR. Article 6 and Article 30 are key for t
aspects.”

2. Also, it's provided under Art. 6 of GDPR that; there are 6 categories of "Lawfulness of Proceeding." However, in this pap
subject to the contents of the parts "Use Restriction" and "Data Declaration" shown on Page 3, only "consent" mode was
mentioned for DAISY proceeding. It becomes necessary for the author to either supplement on it or further clarify the limi
concerning DAISY's function; so as to avoid the possible confusion concerning the coverage of DAISY; viz., for all types of
proceeding? Indeed, subject to the points mentioned here-in-above it is questionable whether the DAISY system may serv
"accountability" function for the "whole" GDPR, as implied from the topic of this paper.

Response:

Additional text has been added to clarify that the consent coding will apply to all legal basis as, even where consent is not
chosen as legal basis, for research projects ethical consent is obtained in almost all cases. These consent conditions shoul
considered as binding if processing is to be fair and in accordance with the information provided at the time of data collect

The compliance with accountability for all chosen legal basis is already explained under “Lawfulness” on page 5.

Specifically, we added the following text to Page 3 and we updated the UML diagram to show the Legal Basis entity in our
model.

“DAISY tracks Legal Bases of ``data processing'' and Use Restrictions per data declaration. Each data declaration can be
associated with one or more legal basis definitions, which in turn is a combination of lawful basis category, out of six as de
by the GDPR, and personal data category i.e. health, genetic, standard personal data etc, as outlined by the GDPR. Even w
consent is not chosen as the legal basis, in most cases ethical consent for the research itself is sought and determines use
conditions that should be seen as much as possible as binding for the continued processing.”

3. It's provided in Art. 30 that the categories of data recipients, transfers of personal data to a third country or an interna
organization need be documented. Obviously, said "transfer" has been articulated in figure 2, however, without correlated
explanation in this regard. In turns, the author is encouraged to supplement on this part in related context so as to compl

the statement.

Response:

An additional sentence had been added on Page 5 to make it clear that the safeguards mentioned referred to the non-EU [illegible] sharing. Specifically, we added the following:

“Such safeguards are an obligation for third country data sharing under the GDPR to ensure that the rights of the data sub[illegible] are retained also under other data protection legislations.”

4. On page 7, the author claims that "Ultimately, full compliance with data protection law may be subject to national or re[illegible] differences depending on the interpretation by the data protection authorities and the GDPR implementations on the differ[illegible] countries through local law." This statement need be further clarified concerning;
(1) Could the wording "different countries" be referred to both the countries within in EU & those outside the region? If so[illegible] compliance requirement could be very different.

Response:

Under bullet “National differences” on Page 7 we clarified that this statement refers to the EEA countries that operate und[illegible] GDPR.

(2) The enactment of GDPR was meant to "uniformly" fill up the "gap" among different member states' personal data prot[illegible] rules. In turns, several basic principles were set to serve as the fundamental rules that all the members must observe. Therefore, when the author stipulates that the full compliance "may be subject to national or regional differences…" Some[illegible] it gives us an impression or an implication that the implementation of GDPR, as a whole, need still be "subject to" the rest [illegible] of legal rules and/or practices of different countries. It's recommended that the author should better make clear the diffe[illegible] between GDPR which is meant to be applied entirely in the EU and an EU Directive which is "up to member countries to de[illegible] how to apply."

Response:

We are currently working on a publication on the different legal implementation of the GDPR as in particular for the subjec[illegible] health and genetic data as well as for research, as well as for several other aspects, there is an enormous scope for nation[illegible] provisions. This topic deserves a longer discussion, in a standalone article, therefore we think it is beyond scope for the D[illegible] paper. However, we have added a brief comment and the reference to another publication that already deals partly with t[illegible] subject.

Specifically, we added the following sentence to “National differences” bullet on Page 7

“In particular in the areas of research and health or genetic data, the GDPR leaves considerable scope for national provisio[illegible] see for example [33].”

Reviewer #2:
An interesting and well-written paper that makes a useful contribution to the thinking and management of issues in biome[illegible] research concerning the GDPR.

I would suggest the following points be considered…

1. Page 7, the section on Integrity, the important point about checksum. The reference 28 is not uninteresting but perhap[illegible] better or additional reference could be the ICH reference to the utility and importance of the MD5 checksum to be found a[illegible] http://estri.ich.org/recommendations/MD5_V1_0.pdf

Response:

We have added a citation to ESTRI recommendation on File Integrity. This has been added as reference number 34.

2. Page 7, The section on confidentiality. There is mention of links to external documents. Might this not be a potential vulnerability? What are the processes available to ensure these links are not broken - and if broken - are repaired?

Response:

We have added a description of planned features of DAISY regarding external links. Additions to Confidentiality bullet on P [illegible] as follows:

“Currently DAISY stores URL links to external documents without checking for erasure or update of those documents. The [illegible] linking feature is intentional as DAISY is typically co-deployed with document management systems dedicated to the stora [illegible] legal documents. We consider such external document management systems to be primarily responsible for preservation [illegible] documents and their revision tracking.”

3. This software is constructed to support compliance with the law. In the pharmaceutical industry, when such software is [illegible] developed it needs to conform with 21 CFR Part 11 which demands "Validation of systems to ensure accuracy, reliability, [illegible] consistent intended performance, and the ability to discern invalid or altered records." https://www.accessdata.fda.gov/scripts/cdrh/cfdocs/cfcfr/CFRSearch.cfm?CFRPart=11&showFR=1&subpartNode=21:1.0.[illegible]

4. Furthermore, when changes are made to the system (a particular vulnerability of open source software) then “Revision [illegible] change control procedures to maintain an audit trail that documents time-sequenced development and modification of sys [illegible] documentation" need to be in place.
5. The financial sector demands similar rigour too as enshrined in the 2002 Sarbanes-Oxley Act

I would suggest just a short paragraph towards the end of the paper that points out that software such as DAISY should b [illegible] installed, tested and managed along the lines of best practices for systems supporting processes in the regulated domain [illegible] have been established in other relevant industries. One example is the pharmaceutical industry and include reference to t [illegible] USA's 21 CFR Part 11, and the EU's Annexe 11 and the importance of computer systems validation and change control.

Response:

These are indeed very good points. We clarified the current and planned features of DAISY in the Integrity bullet on Page [illegible] follows:

“In addition to data and legal documents, another important aspect is the integrity and trustworthiness of metadata, i.e. t [illegible] records in DAISY. Currently DAISY logs all database queries and manipulations, and enforces access control on those operations; but for its records to be used in compliance assessment, more assurances, similar to those provided by Code [illegible] Federal Regulation (CFR) 21 Part 11 complaint software would be necessary. The GDPR is yet to be translated into concre [illegible] industry guidance similar to CFR 21 Part 11 [35], nevertheless in DAISY we plan to implement queryable audit trails of re [illegible] and record signing features in future releases. DAISY’s source code and user manual is tracked in Github, any changes to [illegible] source goes through a process of peer reviewed, approval source changes are fully recorded and source build and test procedures are automated. DAISY should be deployed, tested and managed in protected environments respecting, where applicable, systems support standards and industry best practices, as those in the fi¬nancial or the pharmaceutical sector [illegible]

Editors notes:

In addition, please register any new software application in the bio.tools and SciCrunch.org databases to receive RRID (Research Resource Identification Initiative ID) and biotoolsID identifiers, and include these in your manuscript. This will facilitate tracking, reproducibility and re-use of your tool.

Response:

We registered DAISY mentioned registries, we added the following sentence on Page 8 to denote DAISY’s resource identifi [illegible]

“DAISY has been registered in bio.tools [35] with the identi¬fier biotools:Data_Information_System_DAISY, and in SciCru [illegible] [36] with the resource identi¬fier RRID:SCR_017472”.

Close
