# Supplementary material for: DAISY: A Data Information System for accountability under the General Data Protection Regulation
Source: Gigascience. 2019 Dec 4;8(12):giz140. doi: 10.1093/gigascience/giz140 (PMC6892452; doi:10.1093/gigascience/giz140)
Supplement: giz140_Response_to_Reviewer_Comments_Revision_1 [file giz140_response_to_reviewer_comments_revision_1.pdf]

Author's Response To Reviewer Comments

Close

Dear GigaScience Editors,

We uploaded the final version of our paper titled “DAISY: a Data Information System for accountability under the General Data Protection Regulation”. Specifically, we made the following revisions:

1. In the authors section, we added ORCID identifiers for those authors that are registered with ORCID.

2. In the “methods” section we added “through a web application developed with the Python programming language (RRID:SCR_008394).”

3. In the “availability of source code” section, we added the following:

Project name: Data Information System
Project home page: https://github.com/elixir-luxembourg/daisy
Operating system(s): Platform independent
Programming language: Python 3.6 or higher
Other requirements: Solr 7.x, PostgreSQL 10.x
License: AGPL-3.0
RRID: SCR_017472

4. We added an “availability of data” section with the following paragraph:

“Snapshots of the code are available in the GigaScience GigaDB database [37].”

5. In the references section we added the following:

37. Becker R, Alper P, Grouès V, Munoz S, Jarosz Y, Lebioda J, et al. Supporting data for "DAISY: a Data Information System for accountability under the General Data Protection Regulation". GigaScience Database 2019; http://dx.doi.org/10.5524/100664.

Regards

Pinar ALPER on behalf of authors

Close
