# Supplementary material for: DAISY: A Data Information System for accountability under the General Data Protection Regulation
Source: Gigascience. 2019 Dec 4;8(12):giz140. doi: 10.1093/gigascience/giz140 (PMC6892452; doi:10.1093/gigascience/giz140)
Supplement: giz140_Reviewer_1_Report_Original_Submission — Jui-Chu Lin -- 5/31/2019 Reviewed [file giz140_reviewer_1_report_original_submission.pdf]

Reviewer Report

Title: DAISY: a Data Information System for accountability under the General Data Protection Regulation

Version: Original Submission **Date:** 5/31/2019

Reviewer name: Jui-Chu Lin

Reviewer Comments to Author:

1. For that the research topic specifically highlight on "accountability," it becomes necessary for the author to explain in the part "Background and Motivation" concerning the reason to include Art. 6 and Art. 30 substantially, so that the readers may better catch the points concerning the correlation among different articles.
2. Also, it's provided under Art. 6 of GDPR that; there are 6 categories of "Lawfulness of Proceeding." However, in this paper, subject to the contents of the parts "Use Restriction" and "Data Declaration" shown on Page 3, only "consent" mode was mentioned for DAISY proceeding. It becomes necessary for the author to either supplement on it or further clarify the limitation concerning DAISY's function; so as to avoid the possible confusion concerning the coverage of DAISY; viz., for all types of lawful proceeding? Indeed, subject to the points mentioned here-in-above it is questionable whether the DAISY system may serve the "accountability" function for the "whole" GDPR, as implied from the topic of this paper.
3. It's provided in Art. 30 that the categories of data recipients, transfers of personal data to a third country or an international organization need be documented. Obviously, said "transfer" has been articulated in figure 2, however, without correlated explanation in this regard. In turns, the author is encouraged to supplement on this part in related context so as to complement the statement.
4. On page 7, the author claims that "Ultimately, full compliance with data protection law may be subject to national or regional differences depending on the interpretation by the data protection authorities and the GDPR implementations on the different countries through local law." This statement need be further clarified concerning;

(1) Could the wording "different countries" be referred to both the countries within in EU & those outside the region? If so, the compliance requirement could be very different.

(2) The enactment of GDPR was meant to "uniformly" fill up the "gap" among different member states' personal data protection rules. In turns, several basic principles were set to serve as the fundamental rules that all the members must observe. Therefore, when the author stipulates that the full compliance "may be subject to national or regional differences…" Somehow, it gives us an impression or an implication that the implementation of GDPR, as a whole, need still be "subject to" the restriction of legal rules and/or practices of different countries.

It's recommended that the author should better make clear the difference between GDPR which is meant to be applied entirely in the EU and an EU Directive which is "up to member countries to decide how to apply."

Level of Interest

Please indicate how interesting you found the manuscript: Choose an item.

Quality of Written English

Please indicate the quality of language in the manuscript: Choose an item.

Declaration of Competing Interests

Please complete a declaration of competing interests, considering the following questions:

- Have you in the past five years received reimbursements, fees, funding, or salary from an organisation that may in any way gain or lose financially from the publication of this manuscript, either now or in the future?
- Do you hold any stocks or shares in an organisation that may in any way gain or lose financially from the publication of this manuscript, either now or in the future?
- Do you hold or are you currently applying for any patents relating to the content of the manuscript?
- Have you received reimbursements, fees, funding, or salary from an organization that holds or has applied for patents relating to the content of the manuscript?
- Do you have any other financial competing interests?
- Do you have any non-financial competing interests in relation to this paper?

If you can answer no to all of the above, write 'I declare that I have no competing interests' below. If your reply is yes to any, please give details below.

I declare that I have no competing interests.

I agree to the open peer review policy of the journal. I understand that my name will be included on my report to the authors and, if the manuscript is accepted for publication, my named report including any attachments I upload will be posted on the website along with the authors' responses. I agree for my report to be made available under an Open Access Creative Commons CC-BY license (http://creativecommons.org/licenses/by/4.0/). I understand that any comments which I do not wish to be included in my named report can be included as confidential comments to the editors, which will not be published.

Choose an item.

To further support our reviewers, we have joined with Publons, where you can gain additional credit to further highlight your hard work (see: https://publons.com/journal/530/gigascience). On publication of

this paper, your review will be automatically added to Publons, you can then choose whether or not to claim your Publons credit. I understand this statement.

Yes Choose an item.
