# Supplementary material for: DAISY: A Data Information System for accountability under the General Data Protection Regulation
Source: Gigascience. 2019 Dec 4;8(12):giz140. doi: 10.1093/gigascience/giz140 (PMC6892452; doi:10.1093/gigascience/giz140)
Supplement: giz140_Reviewer_2_Report_Original_Submission — John Wise, MA -- 8/23/2019 Reviewed [file giz140_reviewer_2_report_original_submission.pdf]

Reviewer Report

Title: DAISY: a Data Information System for accountability under the General Data Protection Regulation

Version: Original Submission **Date:** 8/23/2019

Reviewer name: John Wise, MA

Reviewer Comments to Author:

An interesting and well-written paper that makes a useful contribution to the thinking and management of issues in biomedical research concerning the GDPR.
I would suggest the following points be considered…
1. Page 7, the section on Integrity, the important point about checksum. The reference 28 is not uninteresting but perhaps a better or additional reference could be the ICH reference to the utility and importance of the MD5 checksum to be found at: http://estri.ich.org/recommendations/MD5_V1_0.pdf
2. Page 7, The section on confidentiality. There is mention of links to external documents. Might this not be a potential vulnerability? What are the processes available to ensure these links are not broken - and if broken - are repaired?
3. This software is constructed to support compliance with the law. In the pharmaceutical industry, when such software is developed it needs to conform with 21 CFR Part 11 which demands "Validation of systems to ensure accuracy, reliability, consistent intended performance, and the ability to discern invalid or altered records."
https://www.accessdata.fda.gov/scripts/cdrh/cfdocs/cfcfr/CFRSearch.cfm?CFRPart=11&showFR=1&subpartNode=21:1.0.1.1.8.2
4. Furthermore, when changes are made to the system (a particular vulnerability of open source software) then "Revision and change control procedures to maintain an audit trail that documents time-sequenced development and modification of systems documentation" need to be in place.
5. The financial sector demands similar rigour too as enshrined in the 2002 Sarbanes-Oxley Act

I would suggest just a short paragraph towards the end of the paper that points out that software such as DAISY should be installed, tested and managed along the lines of best practices for systems supporting processes in the regulated domain that have been established in other relevant industries. One example is the pharmaceutical industry and include reference to the USA's 21 CFR Part 11, and the EU's Annexe 11 and the importance of computer systems validation and change control.

Level of Interest

Please indicate how interesting you found the manuscript: Choose an item.

Quality of Written English

Please indicate the quality of language in the manuscript: Choose an item.

Declaration of Competing Interests

Please complete a declaration of competing interests, considering the following questions:

- Have you in the past five years received reimbursements, fees, funding, or salary from an organisation that may in any way gain or lose financially from the publication of this manuscript, either now or in the future?
- Do you hold any stocks or shares in an organisation that may in any way gain or lose financially from the publication of this manuscript, either now or in the future?
- Do you hold or are you currently applying for any patents relating to the content of the manuscript?
- Have you received reimbursements, fees, funding, or salary from an organization that holds or has applied for patents relating to the content of the manuscript?
- Do you have any other financial competing interests?
- Do you have any non-financial competing interests in relation to this paper?

If you can answer no to all of the above, write 'I declare that I have no competing interests' below. If your reply is yes to any, please give details below.

'I declare that I have no competing interests'

I agree to the open peer review policy of the journal. I understand that my name will be included on my report to the authors and, if the manuscript is accepted for publication, my named report including any attachments I upload will be posted on the website along with the authors' responses. I agree for my report to be made available under an Open Access Creative Commons CC-BY license (http://creativecommons.org/licenses/by/4.0/). I understand that any comments which I do not wish to be included in my named report can be included as confidential comments to the editors, which will not be published.

Choose an item.

To further support our reviewers, we have joined with Publons, where you can gain additional credit to further highlight your hard work (see: https://publons.com/journal/530/gigascience). On publication of this paper, your review will be automatically added to Publons, you can then choose whether or not to claim your Publons credit. I understand this statement.

Yes Choose an item.
